# Multifaceted Microneedle Patch: A One-Stop Solution to Combat Multitype Wound Infections

**DOI:** 10.34133/bmr.0290

**Published:** 2025-12-19

**Authors:** Hui Xin, Yinghua Xu, Lingling Pan, Shanshan Wang, Bin Li, Ziquan Lv, Xiangjie Yao, Xuan Zou, Xiaobao Jin, Xuemei Lu, Shuiqing Gui

**Affiliations:** ^1^ Intensive Care Unit, Shenzhen Second People’s Hospital, the First Affiliated Hospital of Shenzhen University, Shenzhen 518031, People’s Republic of China.; ^2^ Shenzhen Center for Disease Control and Prevention, Shenzhen 518055, People’s Republic of China.; ^3^ Guangdong Provincial Key Laboratory of Pharmaceutical Bioactive Substances, School of Basic Medical Sciences, Guangdong Pharmaceutical University, Guangzhou Higher Education Mega Center, Guangzhou 510006, People’s Republic of China.; ^4^ Key Laboratory of the Ministry of Health for Research on Quality and Standardization of Biotechnology Products, National Institutes for Food and Drug Control, Beijing 102629, People’s Republic of China.

## Abstract

Bacterial-infected wounds impose a substantial burden worldwide, with polymicrobial infections exacerbating the complexity of healing through dysregulated pH environments and gelatinase-mediated matrix degradation. Herein, we developed a microenvironment-responsive microneedle (MN) patch utilizing a “dynamic warning-graded intervention” strategy. The patch incorporates (a) a bromothymol blue-based pH visual warning system that detects acid-base changes during both acute and chronic infections, (b) a gelatin methacryloyl and exosome matrix material that enables enzyme-triggered release of human bone marrow mesenchymal stem cell-derived exosomes, responding to pathological gelatinase for spatiotemporal drug delivery, and (c) triple therapeutic payloads [hemostasis (halloysite nanotubes)/antibacterial and anti-inflammatory (antimicrobial peptides)/scar reduction (salvianolic acid B)]. In vitro validation demonstrated a bacterial clearance rate exceeding 95% against methicillin-resistant *Staphylococcus aureus*/imipenem-resistant *Pseudomonas aeruginosa*, with biofilm inhibition and disruption rates both surpassing 90%. In vivo experiments demonstrated that MNs showed observable changes in wound color within 8 h in both infectious acute and chronic wounds. In acute wounds, nearly complete healing was achieved within 10 d. By coordinating hemostasis (platelet activation within 60 s), controlling inflammation (62.07% down-regulation of tumor necrosis factor-α), and promoting angiogenesis (2.51-fold up-regulation of CD31), the healing rate of diabetic ulcers was accelerated by 9.20% compared to clinical dressings. This platform provides a foundation for integrating real-time diagnosis and treatment in complex wound management.

## Introduction

Approximately 7.7 million people die from bacterial infections annually, accounting for one-eighth of global deaths, making bacterial infections the second leading cause of mortality [1]. Crucially, polymicrobial synergy [e.g., methicillin-resistant *Staphylococcus aureus* (MRSA)–imipenem-resistant *Pseudomonas aeruginosa* (IRPA) co-colonization] amplifies virulence 10-fold compared to single-species infections through the following mechanisms: pathogenic pH oscillations (acidic glycolysis in the acute phase/alkaline chronic wounds) [2], hyperactive gelatinase [3,4], and biofilm barrier formation [5,6]. Current clinical strategies lack real-time monitoring capabilities and stage-specific interventions, leading to empirical antibiotic overuse and treatment failure rates [7,8].

In recent years, microneedle (MN) technology has emerged as an innovative platform combining minimally invasive characteristics with efficient drug delivery capabilities, demonstrating clinical translational potential in the field of skin wound repair [9,10]. Compared to traditional topical drug administration methods, MNs not only enhance drug bioavailability and reduce systemic toxicity but also enable multi-target synergistic intervention by loading various therapeutic components (such as growth factors and antimicrobial agents), offering new insights into the treatment of complex wounds (such as chronic diabetic ulcers) [11]. However, current research on MN technology remains predominantly focused on static drug delivery modes, which rely on predefined drug release profiles or single treatment interventions, making it challenging to address the differential needs of dynamic pathological stages during wound repair, including inflammation resolution, granulation tissue formation, and epithelial remodeling [12]. Therefore, how to integrate the efficient delivery characteristics of MNs with dynamic sensing functions to construct a closed-loop treatment system that combines “real-time monitoring, intelligent response, and phased intervention” has become a critical direction for breaking through the existing technological bottlenecks [13].

**Fig. 1.**
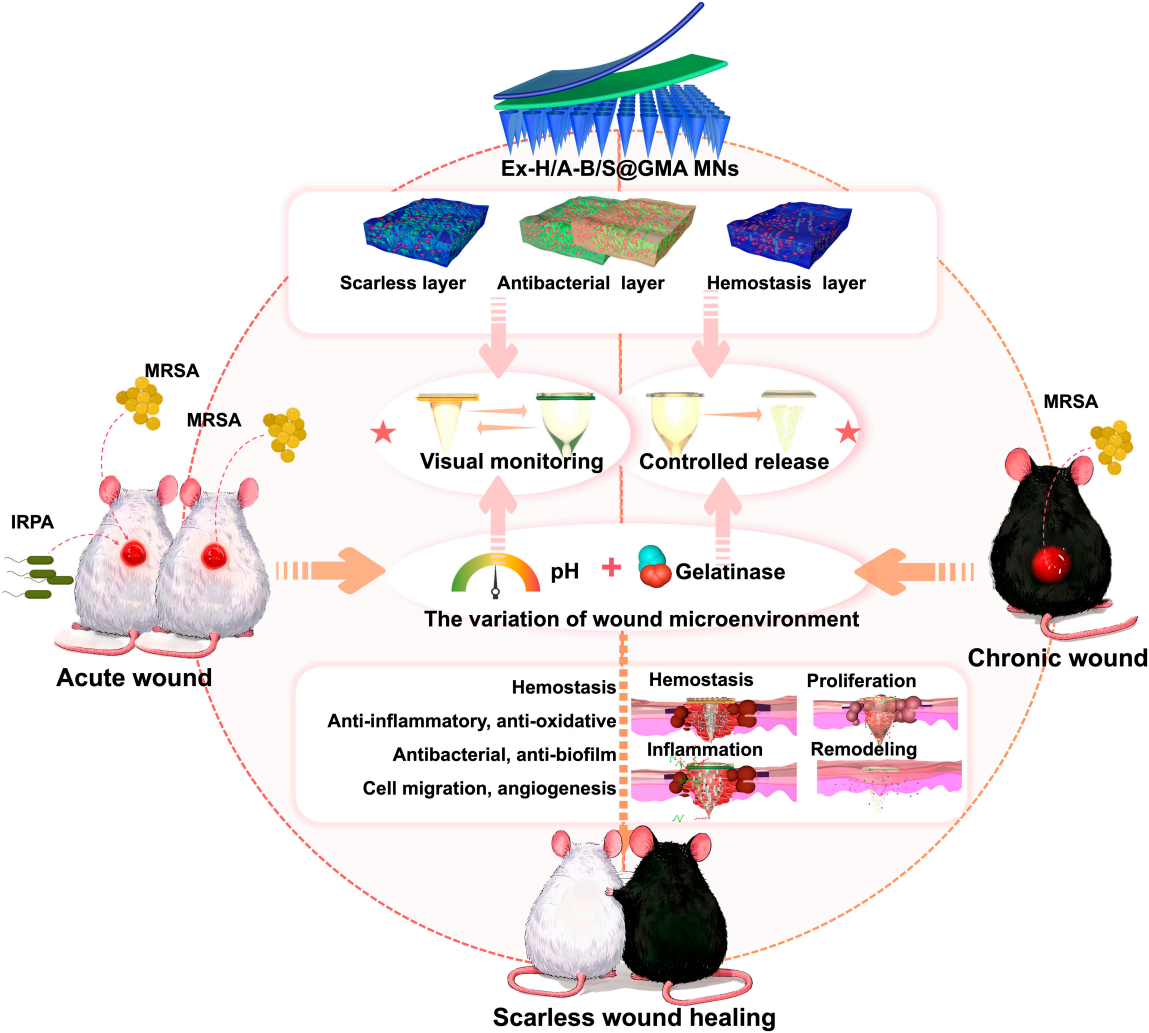
Illustration of the Ex-H/A-B/S@GMA MNs drug delivery platform for scarless wound healing by visual monitoring and controlled release in the whole wound healing process in acute and chronic wounds.

Inspired by “dynamic early warning–stage intervention”, we have developed an MN system utilizing gelatin methacryloyl (GMA). This system consists of 3 primary components: (a) GMA loaded with human bone marrow mesenchymal stem cell-derived exosomes (hBMSCs-Exo, Ex), which serves as the MN matrix material and degrades to release Ex upon gelatinase activation [14–16]; (b) bromothymol blue (BTB), which functions as a pH visual indicator to assess infection status (yellow: pH < 6.0; green: 6.0 ≤ pH < 7.0; blue: pH ≥ 7.0) [17,18]; (c) therapeutic agents, including halloysite nanotubes (HNTs; for hemostasis), antimicrobial peptides (AMPs; exhibiting broad-spectrum antibacterial properties), and salvianolic acid B (SAB; for minimizing scar formation), which modulate the balance of pro-inflammatory and anti-inflammatory factors, thereby promoting scarless wound healing (Fig. 1).

When Ex-H/A-B/S@GMA MNs are applied to wounds, the MN portion rapidly creates microchannels on the wound surface and quickly absorbs water to swell, working synergistically with HNTs to control bleeding. During the inflammatory phase, characterized by infection and the production of gelatinase, GMA swells and degrades, releasing AMPs that effectively clear the infection and inhibit biofilm formation. The presence of BTB in the antibacterial layer serves as a pH-sensitive chromogenic indicator, providing in situ dynamic early warnings of the wound microenvironment’s state due to bacterial infection through visible color changes, which help identify the type and extent of the infection. As the GMA matrix gradually degrades, Ex is slowly released, promoting angiogenesis, cell proliferation, migration, extracellular matrix (ECM) synthesis, and remodeling. Ultimately, this platform is expected to achieve early diagnosis and effective control of infection through precise intervention in the entire wound healing process—including hemostasis, inflammation, proliferation, and remodeling—thereby promoting scar-free wound healing and significantly improving therapeutic outcomes for bacterial-infected wounds, particularly complex wounds such as mixed infections and diabetic ulcers, thus providing a novel and effective strategy for wound management.

## Materials and Methods

### Materials and animals

FK13-a1 (WKRIVRRIKRWLR-NH_2_) and TetraF2W-KK (WWWLKKIW) were obtained from GL Biochem (Shanghai, China). Type B gelatin, HNTs, and Dialysis Membranes Spectra/Por 4 [molecular weight cutoff (MWCO) 12,000 to 14,000] were supplied by Shanghai Yuanye Bio-Technology (Shanghai, China). Ultrapure Milli-Q water (18.2 MW) was used in all experiments and solutions and was stored in a refrigerator at 4 °C. Anti-CD31 (ab28364, Abcam, Cambridge, UK), anti-CD206 (18704-1-AP), anti-α-smooth muscle actin (α-SMA) (ab5694, Abcam, Cambridge, UK), anti-tumor necrosis factor-α (TNF-α) (17590-1-AP), and anti-IL-10 (60269-1-AP) were used. Enzyme-linked immunosorbent assay (ELISA) kits for TNF-α, interleukin-6 (IL-6), IL-1β, and IL-10 were purchased from Jiangsu Meimian Industrial Ltd. (Jiangsu, China). Superoxide dismutase (SOD), catalase (CAT), malondialdehyde (MDA), myeloperoxidase (MPO), aspartate aminotransferase (AST), alanine aminotransferase (ALT), blood urea nitrogen (BUN), and creatinine (CRE) assay kits, along with the H&E stain kit, were procured from Nanjing Jianchen Bioengineering Institute (Nanjing, China). Cell Counting Kit-8 (CCK-8) was purchased from Saint-Bio (Shanghai, China). The calcein-AM/propidium iodide (PI) double staining kit was purchased from Yeasen Biotech (Shanghai, China). *S. aureus* (ATCC 25923), *P. aeruginosa* (GIM 1.535), MRSA (GDMCC 1.644), and IRPA (GDMCC 1.3692) were all purchased from the Guangdong Microbial Culture Collection Center.

BALB/c and C57BL/6 mice (6 to 7 weeks old, 20 to 25 g, male) were obtained from Guangdong Medical Experimental Animal Center. All mice were strictly treated and adhered to the Laboratory Animal Care and Use Guidelines. The Guangdong Pharmaceutical University Experimental Animal Ethics Committee reviewed and approved all animal care protocols and experimental procedures.

### Preparation of GMA

To prepare the GMA solution, first a 10% gelatin solution was made in Dulbecco’s phosphate-buffered saline (DPBS) at 60 °C with stirring. Then, methacrylamide (MA) was added to the gelatin solution at 0.5 ml/min over 4 h, maintaining a temperature of 50 to 60 °C and pH of 7. The reaction was terminated with prewarmed PBS at 40 °C. The mixture was dialyzed in deionized water for 1 week, refreshing the water every 12 h, followed by 24 h of lyophilization. Finally, the lyophilized GMA was dissolved in DPBS at 50 °C and 1% Irgacure 2959 photoinitiator was added.

### Preparation and characterization of Ex-H/A-B/S@GMA MNs

The hemostatic, antibacterial, and scarless layers were sequentially photocrosslinked for 40 s using a 365-nm light-emitting diode (LED) light. The base layer (10% GMA encapsulating Ex and HNTs) served as the hemostatic layer; the antibacterial layer (5% GMA embedding Ex and AMPs) prevented infection; and the top layer (10% GMA containing Ex and SAB) facilitated scarless healing. After crosslinking, the samples were dried and demolded. GMA preparation is detailed in the Supplementary Materials. The degree of gelatin methacrylate (GMA) was determined using ^1^H nuclear magnetic resonance (^1^H NMR) (Bruker Avance NEO 400 MHz, Germany), and its physicochemical structure was analyzed by Fourier transform infrared spectroscopy (FT-IR) (Thermo Scientific iN10, USA). The mechanical strength of the MN patch was assessed via force–displacement curves, and its morphology was observed using scanning electron microscopy (SEM) (Tescan MIRA LMS, Czech Republic). Each experiment was repeated 3 times to ensure result reliability.

### A pilot study on controlled release

GMAs (5%, 10%, and 15% v/v) were immersed in PBS at neutral (pH 7.4) and acidic pH (pH 5.5). The sample’s initial weight (*W*_i_) was measured using a precision electronic scale (JF2004; Yuyao Jinnuo Balance Instrument, China) before immersion in PBS. At each scheduled point, tissues were recorded by the GMA mass (*W*_g_) after removing the surface water. The expansion ratio is determined using the following Eq. (1):

$$\mathrm{Swelling}\;\mathrm{and}\;\mathrm{Degradation}\;\mathrm{ratio}\;(\%)=\frac{W_{g}-W_{i}}{W_{g}}\times 100\%$$ (1)

An appropriate amount of trypan blue was dissolved in 5% and 10% GMA solutions, and the GMA MNs loaded with trypan blue were pressed into artificial skin (made of 1% agar and 7% plant colloid boiled and solidified). The release of trypan blue in the MN was observed under a microscope.

### Visual monitoring and controlled release

The responsiveness of Ex-H/A-B/S@GMA MNs was tested in vitro using BTB with pH buffers and bacterial solutions, measuring absorbance at 570 nm. In vivo, skin wounds were created on mice (infected, diabetic, and control groups), and color changes were recorded over time to track their wound microenvironment-responsive behavior. The study used AMP-RhB and RhB to mimic large and small-molecule drugs, evaluating their release and antibacterial effects through bilayer and MN patch models. AMPs-RhB (0.5 mg/ml) and RhB (0.5 mg/ml) were loaded into different layers. Experiments involved bacterial mixtures [10^6^ colony-forming units (CFU)/ml, 1:1], gelatinase, and PBS with pH 5.5, with AMP-RhB release measured via optical density at 540 nm.

### Computational simulations of Ex-H/A-B/S@GMA MNs

COMSOL Multiphysics (COMSOL, Sverige) simulated drug diffusion in a 3-layer MN system to study skin absorption. It analyzed drug release over 72 h, measuring concentrations at different skin depths. The model comprised 2 primary domains: an MN patch and the skin. The MN patches were further subdivided into 3 components: Ex-S@GMA, Ex-A-B@GMA, and Ex-H@GMA. The stratum corneum, the tough outer layer of highly differentiated cells, is the primary barrier to drug permeation. To better simulate drug permeation, a distinct stratum corneum (20 μm) was included in the skin domain. The model parameters and initial drug concentrations are listed in Table S1. To prevent mass flux across the boundaries, no-flux conditions were applied to the top and bottom surfaces of the model, and symmetric conditions were set along the sides. A physically controlled mesh with fine elements was employed. A time-dependent approach was used to simulate the diffusion of drugs at different skin depths over a total period of 72 h with a time step of 2 h. Drug concentrations in 3 parallel planes were assessed: plane 1 (upper), located 300 μm beneath the skin surface (halfway through the MN); plane 2 (middle), at 600 μm (at the MN tip); and plane 3 (lower), at 1,000 μm, corresponding to the dermis layer.

### Antimicrobial properties in vitro

To test the antimicrobial properties against MRSA and IRPA, bacteria were cultured in Luria–Bertani (LB) broth and exposed to various samples (PBS, Ex-HNTs@GMA, AMPs@GMA, SAB@GMA, Ex-H/A/S@GMA, and VAN) for 24 h. Bacterial growth was measured at OD_600 nm_, and antimicrobial efficacy was determined by counting the CFU on Mueller–Hinton plates after 12-h incubation. A live/dead bacterial staining kit (40747ES76, Yeasen Biotechnology) was used to compare the antimicrobial abilities.

### Biofilm clearance experiment

MRSA biofilms were formed in LB medium for 24 h, treated with MN patches for 12 h, and then washed and fixed with methanol. Crystal purple staining was used to measure biofilm clearance at 570 nm after dissolving in 33% glacial acetic acid. Additionally, SYTO 9 staining was applied for laser confocal imaging.

### Blood compatibility

The blood compatibility of various MN samples was assessed using hemolysis assays, with PBS and 0.1% Triton X-100 as controls. Red blood cell (RBC) suspensions (5%, v/v) were incubated with the samples and controls at 37 °C for an hour, and then absorbance at 540 nm was measured to calculate hemolysis rates. The hemolytic rate was calculated using Eq. (2):

$$\text{Hemolysis rate}\;\left(\%\right)=\frac{A_{m}-A_{n}\;}{A_{p}-A_{n}}\times 100\%$$ (2)

where *A*_m_, *A*_n_, and *A*_p_ represent the absorbance values of the MN, negative control, and positive control groups, respectively.

### Hemostatic potential in vitro and in vivo

The blood clotting index (BCI) evaluated the hemostatic potential of various MN patches in vitro. After prewarming samples at 37 °C, whole blood and clotting reagents were added. Clotting was monitored at 3 and 7 min, with absorbance measured at 540 nm to calculate BCI using Eq. (3):

$$\mathrm{BCI}\;(\%)=\frac{OD{\;}_{\text{sample}}}{OD{\;}_{\text{reference}}}\times 100\%$$ (3)

Each sample was repeated thrice (*n* = 3).

The hemostatic ability of Ex-H/A/S@GMA was tested using 2 mouse models: liver hemorrhage and tail amputation, each with 6 mice. In the liver hemorrhage model, a 2-mm bleeding hole was created and treated with Ex-H/A/S@GMA. In the tail model, the tail was clipped and similarly treated. Blood loss was measured using filter paper, calculating the difference in weight before (*W*_0_) and after (*W*_1_) treatment as (*W*_1_ − *W*_0_).

### Anti-inflammatory effects

The anti-inflammatory effects were evaluated using nitric oxide and cytokines as indicators. RAW264.7 cells were grown in a 96-well plate, stimulated with lipopolysaccharide (LPS) (10 μg/ml) to provoke an inflammatory response, and incubated for 4 h. Following treatments with samples for 24 h, nitrite levels were measured using a nitric oxide detection kit (Beyotime, Shanghai, China), and immunofluorescence assessed RAW264.7 secretion of TNF-α and IL-10.

### Cell migration test

Cell migration was assessed using scratch and Transwell assays in NIH-3T3 and HaCaT cells. For the scratch assay, cells were imaged at post-scratch intervals, and wound healing was quantified using ImageJ software (GraphPad Software Inc., USA). In the Transwell assay, cells were added to the upper chamber of a 24-well plate (Corning, NY, USA) in serum-free medium and treated with different samples. Cell migration was quantified using the ImageJ software after imaging with a microscope.

### Construction of a co-infected full-thickness wound model

Ex-H/A-B/S@GMA MNs were evaluated in vivo using a co-infected full-thickness wound model to assess scarless wound healing and antimicrobial efficacy. After anesthesia with 60 mg/kg pentobarbital, 2 circular 8-mm-diameter full-layer skin wounds were inflicted on the back of each mouse using sterile surgical scissors. The mixed bacterial solution (100 μl, 1 × 10^8^ CFU/ml, MRSA and IRPA, ratio 1:1) was injected into the full-thickness wounds. The mice were then randomly divided into 4 groups (*n* = 6): the Model group (treated with 100 μl of PBS), GMA MNs group, Ex-H/A-B/S@GMA MNs group, and a positive control group using 3M Tegaderm Film (3M Health Care, USA). Photographs were systematically captured to monitor the progression of wound healing, and ImageJ software was used to quantify wound closure.

### Wound healing evaluation in co-infected mice

After surgery, mice were sacrificed on days 3, 7, and 14. Inflammatory factors were observed on day 3, whereas epithelial length and thickness, granulation and dermal tissue thickness, number of follicles, and collagen deposition were evaluated on days 7 and 14 using hematoxylin and eosin (H&E) and Masson’s trichrome staining. The presence of inflammatory cells, angiogenesis, and fibroblasts in the wound was detected using immunofluorescence staining. Additionally, levels of TNF-α, IL-10, pro-reparative M2 phenotypic markers (CD206), CD31, and α-SMA were assessed. ELISA (Jiangsu Meimian Industrial) was employed to measure the levels of TNF-α, IL-10, IL-1β, and IL-6. The expression levels of vascular endothelial growth factor (VEGF), CD31, collagen I (Col I), and Col III were quantified using quantitative polymerase chain reaction (qPCR). The calculation of wound closure is shown in Eq. (4):

$$\text{Wound closure}\;\left(\%\right)\;=\;\frac{S_{0}-S_{t}}{S_{0}}\times 100\%$$ (4)

where *S*_0_ and *S_t_* represent the wound area on day 0 and day *t* (*t* = 3, 7, 10, 14), respectively.

### Construction of infected full-thickness diabetic wound model

Male C57BL/6 mice were intraperitoneally injected with streptozotocin (STZ; Macklin, China) for 5 consecutive days. Diabetic models were successfully established when the glucose levels of the mice consistently exceeded 16.7 mM. Full-thickness wounds (8-mm diameter) were created on the back of each mouse using a biopsy punch. A volume of 100 μl of MRSA was repeatedly injected into the skin defect wound. The wounds were sequentially treated with GMA, Ex-H/A-B/S@GMA MNs, and Film. The wound areas were observed, and pictures were taken on days 0, 3, 7, and 14 after surgery.

### Wound healing evaluation in diabetic mice

Skin tissues were harvested on postoperative days 3, 7, and 14 for H&E staining. The antibacterial effects of the materials were evaluated on days 0, 3, 7, and 14. Giemsa staining was performed on days 3 and 7 to verify biofilm formation. TNF-α, IL-1β, IL-6, and IL-10 levels were measured in a 10% skin homogenate using ELISA kits. Immunofluorescent staining for TNF-α and IL-10 was conducted on days 7 and 14 to assess the inflammatory response. Immunohistochemical staining was performed using α-SMA antibodies. Masson’s trichrome staining was used on day 14 to evaluate wound closure and collagen formation. Quantitative reverse transcription PCR (qRT-PCR) was performed to detect gene expression. MDA, CAT, SOD, and MPO levels were measured in a 10% skin homogenate. ROS levels in the wound bed and surrounding skin were detected using a staining kit (Sangon Biotech, Shanghai, China).

### Biocompatibility in vitro and in vivo

The cytotoxicity of MN extracts was tested on NIH-3T3, HaCaT, and L929 cells using CCK-8 and Live/Dead assays. Cells were treated with extracts (Ex-HNTs@GMA, AMPs@GMA, SAB@GMA, Ex-H/A/S@GMA) in 96-well plates (for CCK-8) or 24-well plates [for Live/Dead staining (Sigma-Aldrich, USA)]. Cell viability was calculated using Eq. (5), and each experiment was done in triplicate.

$$\text{Cell viability}\;\left(\%\right)=\frac{OD{\;}_{\text{treatment}}-OD{\;}_{\text{blank}}\;}{OD{{\;}_{\text{control}}}_{}-OD{\;}_{\text{blank}}}\times 100\%$$ (5)

where OD treatment, OD control, and OD blank refer to the uptake of the MN extract treatment, nontoxic treatment, and cell-free medium groups, respectively.

In vivo, the toxicity of Ex-H/A-B/S@GMA MNs was determined after 14 d. H&E staining of the major organs (liver, heart, spleen, lungs, and kidneys) was performed. Serum liver function (ALT, AST) and kidney function (BUN, CRE) were measured.

## Results and Discussion

### Preparation and characterization of Ex-H/A-B/S@GMA MNs

**Fig. 2.**
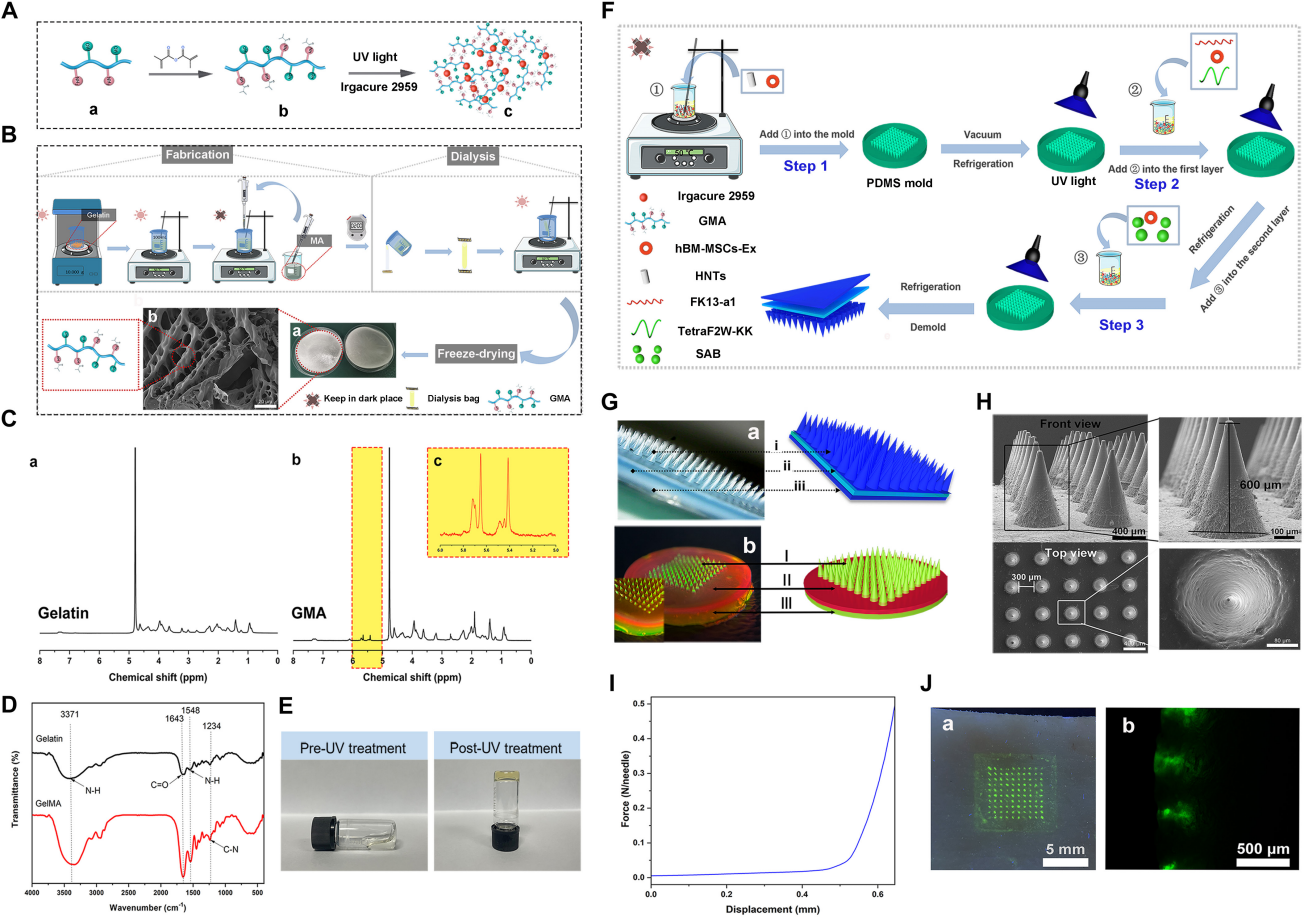
Preparation and characterization of Ex-H/A-B/S@GMA MNs. (A) Schematic showing the synthesis and photo-crosslinking of GMA. (B) Diagram of GMA synthesis. (C) ^1^H NMR of gelatin and GMA. (D) Fourier transform infrared spectra of gelatin and GMA. (E) Photograph of pre-UV treatment GMA and post-UV treatment GMA. (F) Schematic of the preparation of Ex-H/A-B/S@GMA MNs. (G) Left photographs were captured, and the right images were generated using MAXON Cinema 4D. (H) SEM images of Ex-H/A-B/S@GMA MNs. (I) Mechanical compression tests of the Ex-H/A-B/S@GMA MNs. (J) Images after application to porcine cadaver skin. (a) Fluorescence micrographs of MNs-treated skin after being inserted with FITC@GMA MNs. (b) Green fluorescence indicated the implanted FITC@GMA MNs.

The preparation of GMA is presented in Fig. 2A and B. The porous structure inside the GMA is shown in a freeze-dried photograph of the GMA and SEM. Figure 2C shows the degree of MA conjugation by ^1^H NMR, and the synthesized GMA was found to be 69% methacrylated. Additionally, Fig. 2D and E demonstrates the successful synthesis of GMA as confirmed by FT-IR analysis and photographs of the changes before and after ultraviolet (UV) treatment; the absorption peaks of N–H bending and C–N stretching (amide III) at 1,548 and 1,234 cm^−1^, respectively, are ascribed to N–H bending coupled to C–N stretching (amide II) [19,20]. Due to the N–H stretching vibration, a typical absorption peak originated around 3,371 cm^−1^ [21]. At 1,643 cm^−1^, the absorption band was assigned to the C=O stretching (amide I) vibration [22]. Energy-dispersive x-ray spectroscopy (EDS) further highlighted the presence and homogeneous distribution of HNTs within the GMA (Fig. S1). As shown in Fig. S2, Ex was isolated and purified via ultracentrifugation and a round spherical morphology and diameter were observed. In our previous study, we successfully isolated and purified hBM-MSC-Ex and utilized them for the targeted delivery of therapeutic agents [23,24]. Live imaging experiments were conducted to validate the potential of the GMA MNs to enhance the stability of Ex vivo. Fluorescence intensity was measured before and after 30 min (Fig. S3).

The preparation process for the Ex-H/A-B/S@GMA MNs is schematically displayed in Fig. 2F. Hemostatic (*i*/I), antibacterial (*ii*/II), and scarless layers (*iii*/III) are shown in Fig. 2G, and SEM results showed that the height of MNs and the substrate-to-substrate distance of adjacent MNs are 600 and 300 μm, respectively (Fig. 2H). The mechanical properties of MNs are fundamental to successful skin insertion [19]. Figure 2I shows that each needle could withstand more than 0.1 to 0.2 N of compressive forces, strong enough to achieve successful skin puncture. We further tested the ability of MNs to penetrate the skin using a porcine cadaver skin model. After applying the FITC (fluorescein isothiocyanate)@GMA MNs, a complete array of green spots was visible on the porcine cadaver skin, demonstrating that the MNs were successfully inserted into the skin throughout the epidermis and remained in the dermal layer (Fig. 2J). The safety of skin application is a crucial aspect to consider. As displayed in Fig. S4, after removing the Ex-H/A-B/S@GMA MNs from the skin, the microchannels formed by the MNs in the mouse carcasses remained for 5 min before gradually becoming lighter. After 30 min, the peak disappeared. These results revealed no skin irritation, such as edema or erythema, within 1 h of MN penetration, compared to 1.5% formol.

### Visual monitoring

**Fig. 3.**
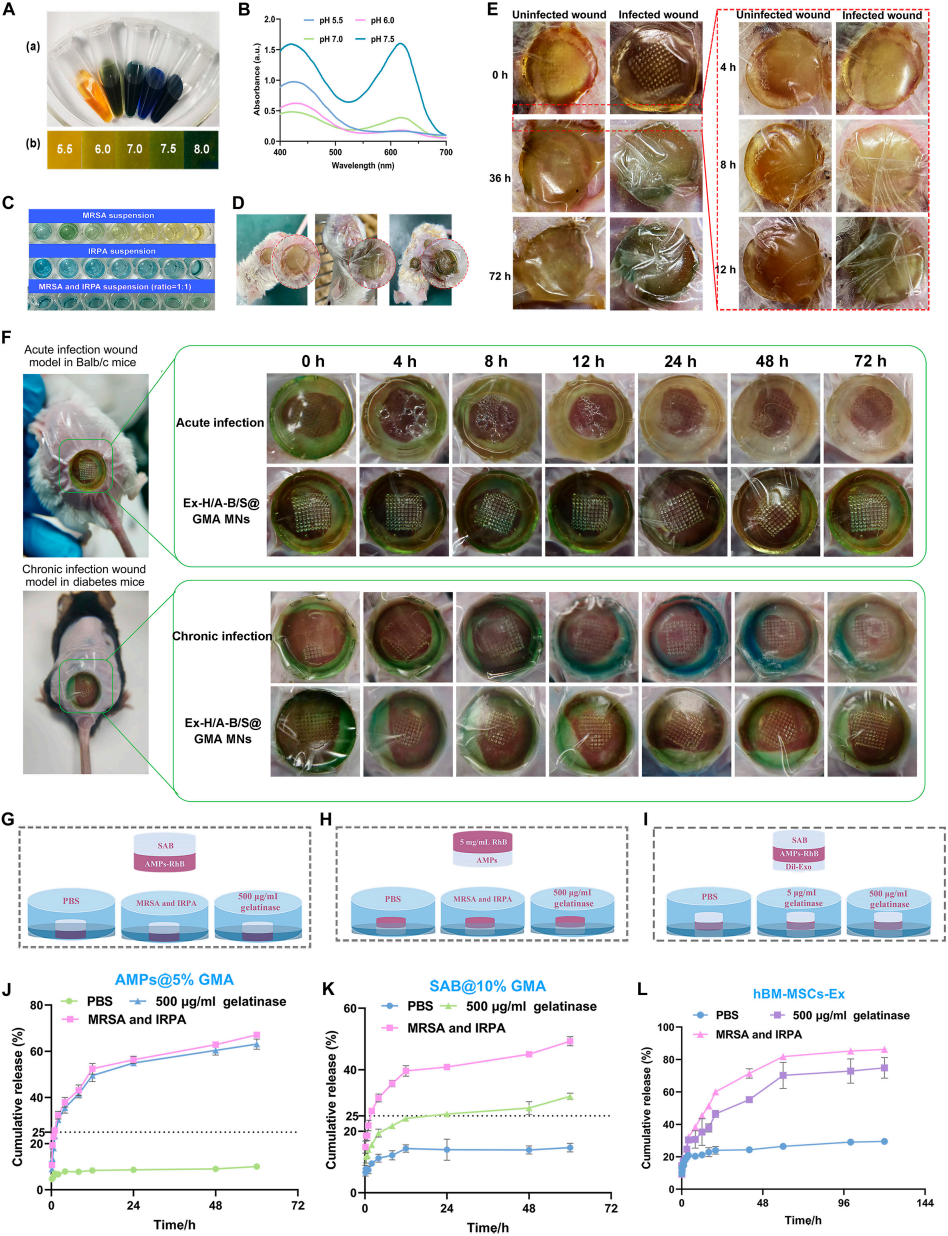
Visual detection ability and drug release studies of Ex-H/A-B/S@GMA MNs. (A) (a) Color change and (b) pH test paper of 0.1% BTB at pH 5.5, 6.0, 7.0, 7.5, and 8.0. (B) UV–vis absorption spectra of BTB at different pH. (C) Different bacteria suspensions and concentrations are detected using 0.1% BTB solution. (D) Schematic of diagnosis bacteria-infected wound model of mice. (E) The color change over time was compared between infected and uninfected wounds in vivo. (F) Color change over time in acute and chronic wound models. (G) Two-layer model of the release of AMPs. (H) Two-layer model of the release of SAB. (I) Three-layer structure model of release behavior of hBM-MSC-Ex. (J) Release behavior of AMPs in PBS, mixed suspension (MRSA and IRPA), and 500 μg/ml gelatinase. (K) Release behavior of SAB. (L) Release behavior of hBM-MSC-Ex. *n* = 3.

As shown in Fig. 3A, as the pH increases from approximately 5.5 to 6, the color of the BTB solution shifts from yellow to green. Upon further increasing the pH to 7.5, the color transitioned from green to blue, and the UV–visible (UV–vis) absorption spectra of BTB correspondingly reflected these color changes across the pH range of 5.5 to 7.5 (Fig. 3B). When MRSA and/or IRPA solutions were mixed with the BTB solution, the color changed in various ways (Fig. 3C). As a color indicator, BTB can endow MN patches with pH sensitivity [25], detect the microenvironmental status of bacterial infections in situ via visible color changes, and initiate treatment measures as soon as possible.

Figure 3D and E shows the difference in color change of Ex-H/A-B/S@GMA MNs between uninfected and infected wounds from 0 to 72 h in MRSA-and IRPA-infected mouse acute wound models, which may be due to the acidic microenvironment resulting from lactic acid secretion, causing pyocyanin, a toxic metabolite derived from *P. aeruginosa*, to turn green [26,27]. Differently, in the acute wound model infected with MRSA, the GMA MNs change from green to yellow, while in the chronic wound model, they shift from green to blue. After treatment, the wound becomes weakly alkaline with a pH of 7, which is conducive to healing and shows that BTB retains visual monitoring capabilities in Ex-H/A-B/S@GMA MNs and can alert to various wound infections (Fig. 3F).

### Controlled release

As shown in Fig. S5A and B, the maximum swelling rate of 5% GMA was 350% to 400%, and it started to degrade gradually after 20 h, which was used as an “antibacterial” layer to release AMPs for rapid sterilization and to pass through the inflammatory phase of wound healing as rapidly as possible. The swelling and degradation rate of 10% GMA is lower than that of 5% GMA, but it can be maintained at 200% to 300%, which may be attributed to the differences in pore structure and drug diffusion kinetics caused by varying crosslinking densities. We speculate that the rapid release of 5% GMA may correspond to the high drug concentration required for early wound healing, while the stable release of 10% GMA may be more suitable for long-term treatment. The MN layer (10% GMA) can quickly form microchannels, thereby facilitating the release of the antibacterial layer (5% GMA) at the location of the skin dermis (Fig. S5C).

Individual release behaviors of AMPs/SAB/hBM-MSC-Ex and morphological evolution of the triple-layer structure were analyzed using 4 customized models (Fig. 3G to L and Fig. S6A). The cumulative release rates at 24 h reached nearly 60% for AMPs, approximately 40% for SAB, and over 60% for hBM-MSC-Ex, which was co-released from the hemostatic and antibacterial layers, synergizing with AMPs/SAB to accelerate rapid wound healing. To deepen our understanding of the experimental outcomes, computational simulations were performed using the COMSOL Multiphysics software (COMSOL Inc.). The model parameters and initial drug concentrations are listed in Table S1. The CAD (computer-aided design) drawings of the MN components are shown in Fig. S6C, depicting the various regions of the model, the complete MN array morphology, and detailed views of the individual MNs from the front, top, and side perspectives. Figure S6D and E qualitatively demonstrates the differences in drug release rates with and without MNs. The application of MNs accelerated the drug release into the dermis, as evidenced by color coding and annotations. The 3-dimensional (3D) models depicted in Fig. S6F to I, along with Fig. S6J to L, show the individual drug loading on each layer of the MN patch and the drug release at the 3 different planes (300, 600, and 1,000 μm). Initially, Ex was evenly distributed across the wound area after 48 h, with the released amount stabilizing, indicating a synergistic effect with GMA on hemostasis within the first 0 to 2 h. Subsequently, the cumulative release of AMPs peaked at 24 h and then stabilized, which was beneficial for swiftly transitioning through the inflammatory phase and inhibiting bacterial growth, leading to a proliferative remodeling phase. As a small molecule, the efficiency of SAB in penetrating the dermis layer was significantly enhanced by the MN-formed microchannels, and it worked in conjunction with Ex to promote wound healing in the early stages while inhibiting excessive wound fibrosis and preventing scar formation in the later stages.

### Antibacterial and anti-biofilm properties in vitro

**Fig. 4.**
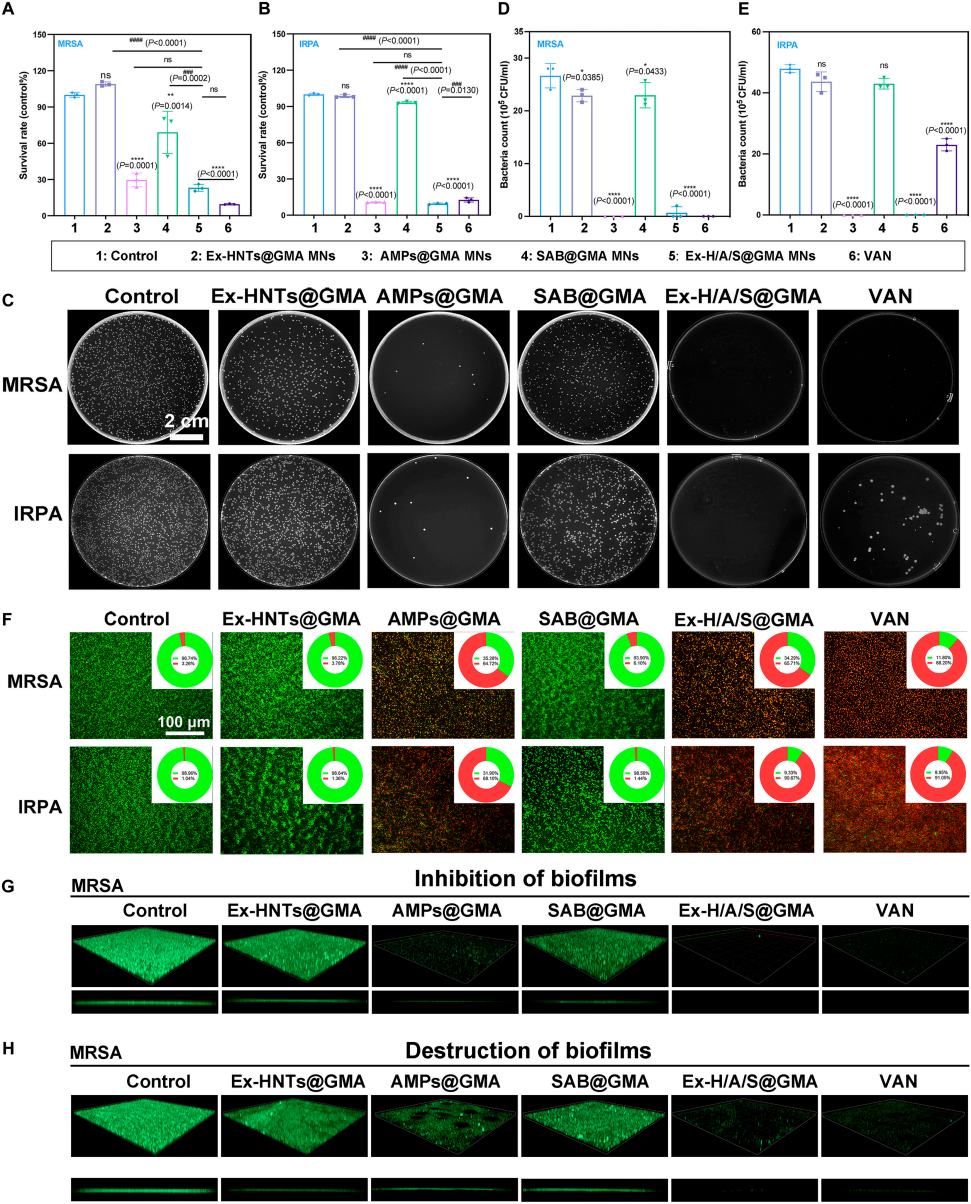
Antibacterial and anti-biofilm effect of the Ex-H/A-B/S@GMA MNs in vitro. (A and B) Survival rate. (C) Images of clones of survival bacteria in different groups. (D and E) Plate colonies were counted for MRSA and IRPA. (F) Live/dead staining. (G and H) Inhibition and destruction of MRSA biofilm following treatment with various groups. *n* = 3. An asterisk (*/^#^) indicates a statistically significant difference (*P < 0.05, **P < 0.01, ****P < 0.0001, *^###^P* < 0.001, *^####^P* < 0.0001).

As depicted in Fig. 4A and B, the results showed enhanced antibacterial efficacy with AMPs present. As shown in Fig. 4C to F, in the agar plate and live/dead staining results, significant bacterial death was observed in the AMPs@GMA and Ex-H/A/S@GMA groups. The inhibitory effects of different samples on MRSA biofilms were also investigated. As shown in Fig. S7, Ex-H/A-B/S@GMA MNs damaged the initial biofilm and mature biofilm of MRSA to different degrees, which were 83.5% and 75.6%, respectively, whereas vancomycin had similar destructive effects. For the IRPA of initial and mature biofilms, the Ex-H/A-B/S@GMA MNs were superior to the vancomycin group. Moreover, as shown in Fig. 4G and H, the confocal laser results showed that the Ex-H/A-B/S@GMA MNs inhibited and destroyed anti-biofilm properties. The destructive effect of Ex-H/A-B/S@GMA MNs on biofilm was like that of the vancomycin group.

### Hemostatic, anti-inflammatory, anti-oxidative, and wound healing properties of Ex-H/A-B/S@GMA in vitro

**Fig. 5.**
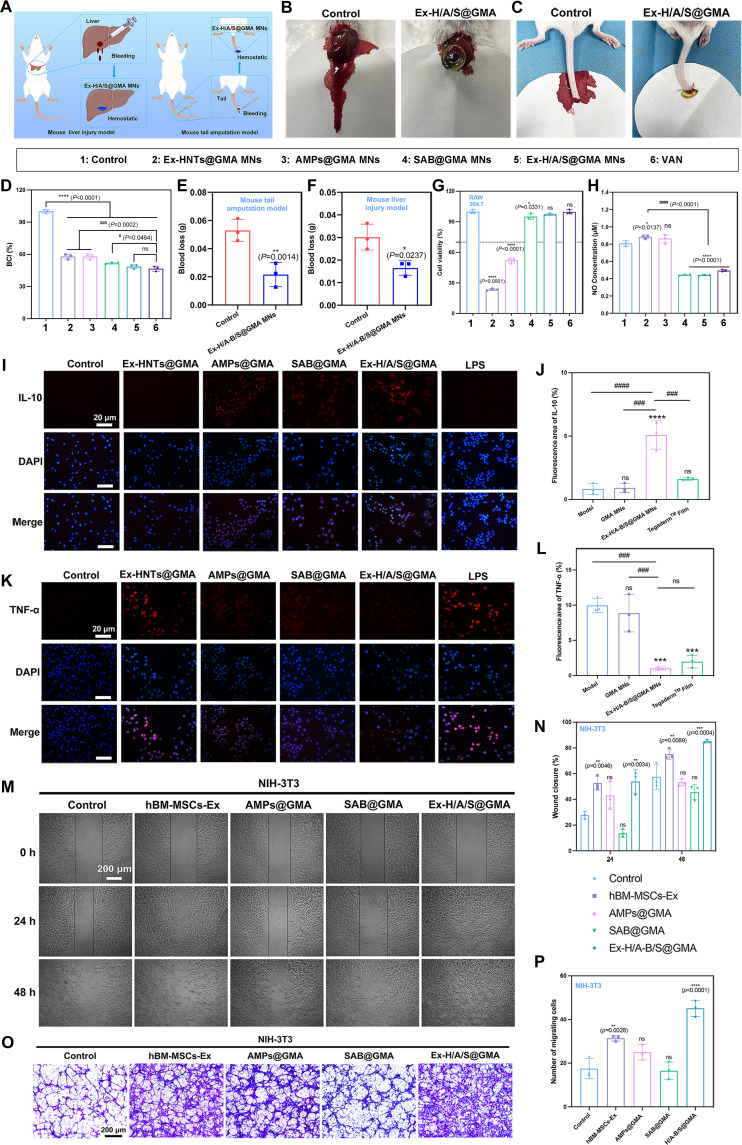
Hemostatic, anti-inflammatory, and cell migration ability of the Ex-H/A-B/S@GMA MNs in vitro. (A) Schematic of hemostatic properties of the Ex-H/A/S@GMA MNs. (B) Loss of blood in the hemorrhaging hepatic mouse model and (E) statistical outcome. (C) Loss of the blood in the mouse tail amputation model and (F) statistical results. (D) In vitro whole BCI. (G) RAW264.7 cell viability. (H) NO production. (I) Expressions of IL-10 and (J) statistical outcome. (K) TNF-α detected by immunofluorescent staining and (L) statistical outcome. (M and N) Photographs and quantitative results of NIH-3T3 cell migration over time. (O and P) Transwell migration assays and quantitative analysis of the number of migratory cells were performed on NIH-3T3 cells with different treatments. *n* = 3. An asterisk (*/^#^) indicates a statistically significant difference (*P < 0.05, **P < 0.01, ***P < 0.001, ****P < 0.0001, *^###^P* < 0.001, *^####^P* < 0.0001).

The whole BCI is often used to assess blood coagulation [28]. The BCI scores of the HNTs@GMA and Ex-H/A-B/S@GMA groups decrease to 48.5% and 46.6%, respectively, rather than the PBS group. The treatment of Ex-H/A-B/S@GMA MNs led to reductions in blood loss of 43.7% and 60%, in the mouse liver injury model and tail amputation, respectively. These results may be attributed to the synergistic effect of GMA and HNTs on hemostasis owing to their microporous structure and good RBC adhesion (Fig. 5A to F).

CCK-8 and live/dead cell staining images revealed that cell viability was not affected, suggesting that Ex-H/A-B/S@GMA MNs had negligible cytotoxicity toward NIH-3T3, HaCaT, and L929 cells (Fig. S8). The CCK-8 assay showed that the survival rate of RAW264.7 significantly increases by 75.5% in Ex-H/A-B/S@GMA compared to the LPS group, probably because of the AMPs/SAB-mediated reduction in macrophage inflammatory responses (Fig. 5G). The nitric oxide (NO) levels were reduced by 50.0%, 47.7%, and 44.3% in AMPs@GMA, SAB@GMA, and Ex-H/A-B/S@GMA groups (Fig. 5H). Moreover, IL-10 is a cytokine with anti-inflammatory activity, whereas the proinflammatory cytokine TNF-α contributes to inflammatory diseases [29]. A marked up-regulation of IL-10 and a simultaneous down-regulation of TNF-α in Ex-H/A-B/S@GMA MNs-treated cells versus the LPS group was observed (Fig. 5I to K).

Excessive free radicals in chronic wound sites often cause intense oxidative stress and delay wound healing [30]. Therefore, wound dressings with free-radical-eliminating properties are beneficial for wound healing. The DPPH (1,1-diphenyl-2-picrylhydrazyl) scavenging capacity of the hydrogels is shown in Fig. S9A. Ex-H/A-B/S@GMA showed excellent antioxidant effects caused by SAB in the MNs. Next, NIH-3T3 cells were treated with H_2_O_2_ to construct an oxidative stress model. As shown in Fig. S9B, the oxidative stress model was successfully constructed, and the cell viability was 40% to 60% at 300 μM. Subsequently, NIH-3T3 cells were treated differently at 300 μM H_2_O_2_; the cell survival rate of the Ex-H/A-B/S@GMA group was over 75%, which proved that Ex-H/A-B/S@GMA has a protective effect on cells and excellent antioxidant properties (Fig. S9C). SOD repairs cells and reduces damage from peroxides (the most common free radicals in the body). The SOD activities in the Ex-H/A-B/S@GMA and SAB@GMA groups were significantly higher than those in the other groups, further proving that AMPs and SAB had excellent antioxidant effects (Fig. S9D). As shown in Fig. 5M to P and Fig. S10, the treatment of Ex-H/A/S@GMA increases the wound closure rate, as indicated by the scratch assays, from 57.5% to 86.5% in NIH-3T3 cells and 33.7% to 80.5% in HaCaT cells. Similarly, following the treatment Ex-H/A/S@GMA, the number of migrating cells exhibited a 1.57- and 1.60-fold increase relative to the control group in NIH-3T3 and HaCaT cells, respectively.

### Wound healing evaluation of Ex-H/A-B/S@GMA in co-infected mice

**Fig. 6.**
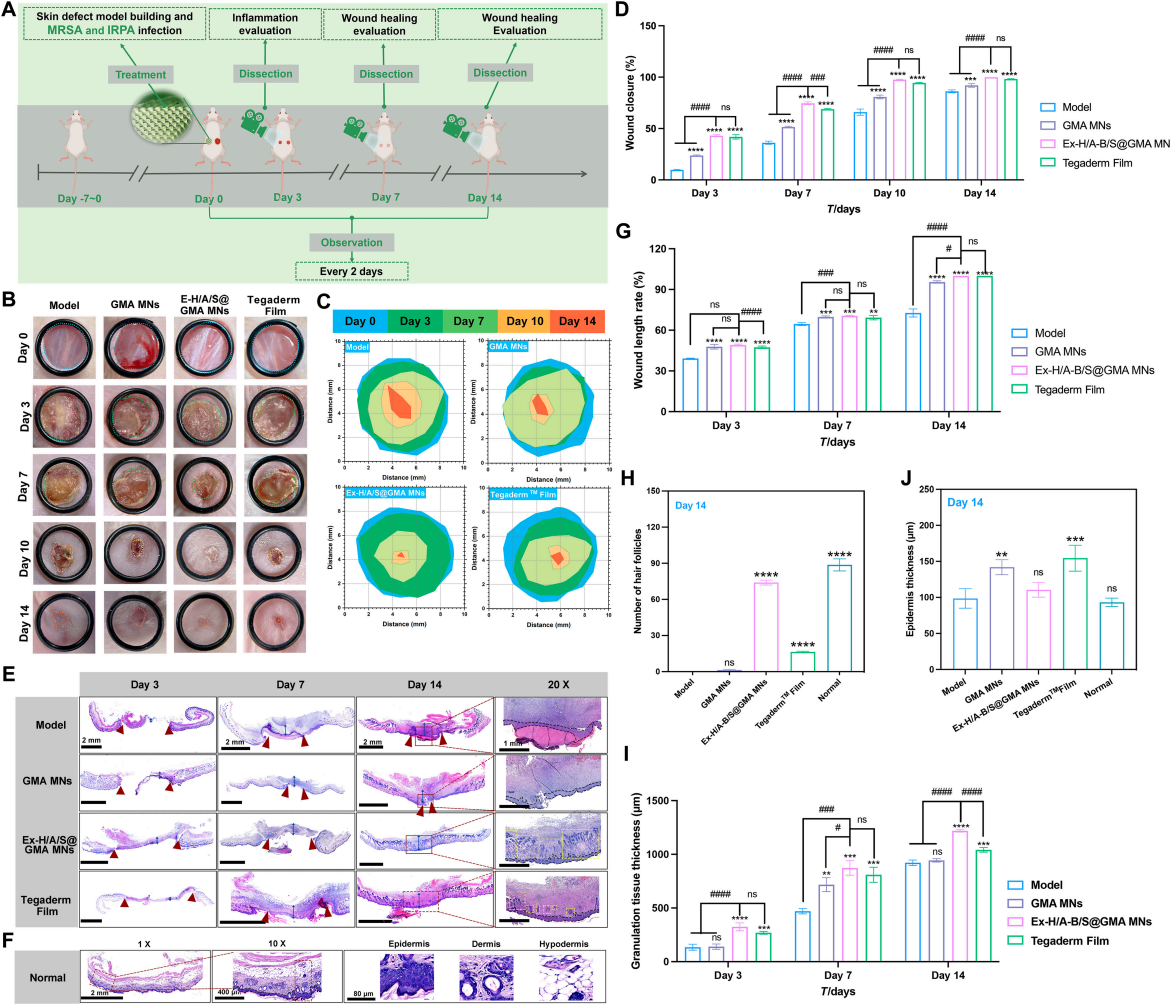
Wound healing evaluation of Ex-H/A-B/S@GMA MNs in co-infected mice. (A) Schematic depicting the timeline of the experimental procedure. (B) Representative photos of wounds treated with different specimens. (C) Diagrammatic images of wound contraction. (D) Quantitative wound closure outcomes. (E) Histological micrographs of H&E-stained sections. Red arrows indicate the length of the wound. Blue 2-way arrows indicate the thickness of the granulation tissue. Black spots represent epidermis thickness. Yellow box means hair follicles. (F) H&E-stained normal skin. (G) Wound length rate. (H) Granulation tissue thickness. (I) Number of follicles. (J) Epidermal epidermis thickness. *n* = 6. An asterisk (*/^#^) indicates a statistically significant difference (**P < 0.01, ***P < 0.001, ****P < 0.0001, *^#^P* < 0.05, *^###^P* < 0.001, *^####^P* < 0.0001).

Figure 6A shows the construction and treatment processes of the infected full-thickness wound model. As shown in Fig. 6B to D, the images of the wound site revealed that Ex-H/A-B/S@GMA MNs accelerated wound closure faster than the other groups, with a wound healing rate of more than 70% by day 7 and almost complete healing by day 10. More importantly, less scar tissue was observed in the Ex-H/A-B/S@GMA MNs group than in other groups. As shown in Fig. 6E to I, the hematoxylin and eosin (H&E) staining results demonstrated that the wound healing rate of the Ex-H/A-B/S@GMA MNs group reached 100%, with the number of hair follicles and the thickness of the epithelium being comparable to those of the normal group. Granulation tissue fills the open space of a full-thickness wound, supplies blood, and helps prevent infection. The granulation tissue thickness of the Ex-H/A-B/S@GMA MNs group increases by 24.4%, compared to the model group.

### Antibacterial, anti-inflammatory, and scarless wound healing of Ex-H/A-B/S@GMA in co-infected mice

**Fig. 7.**
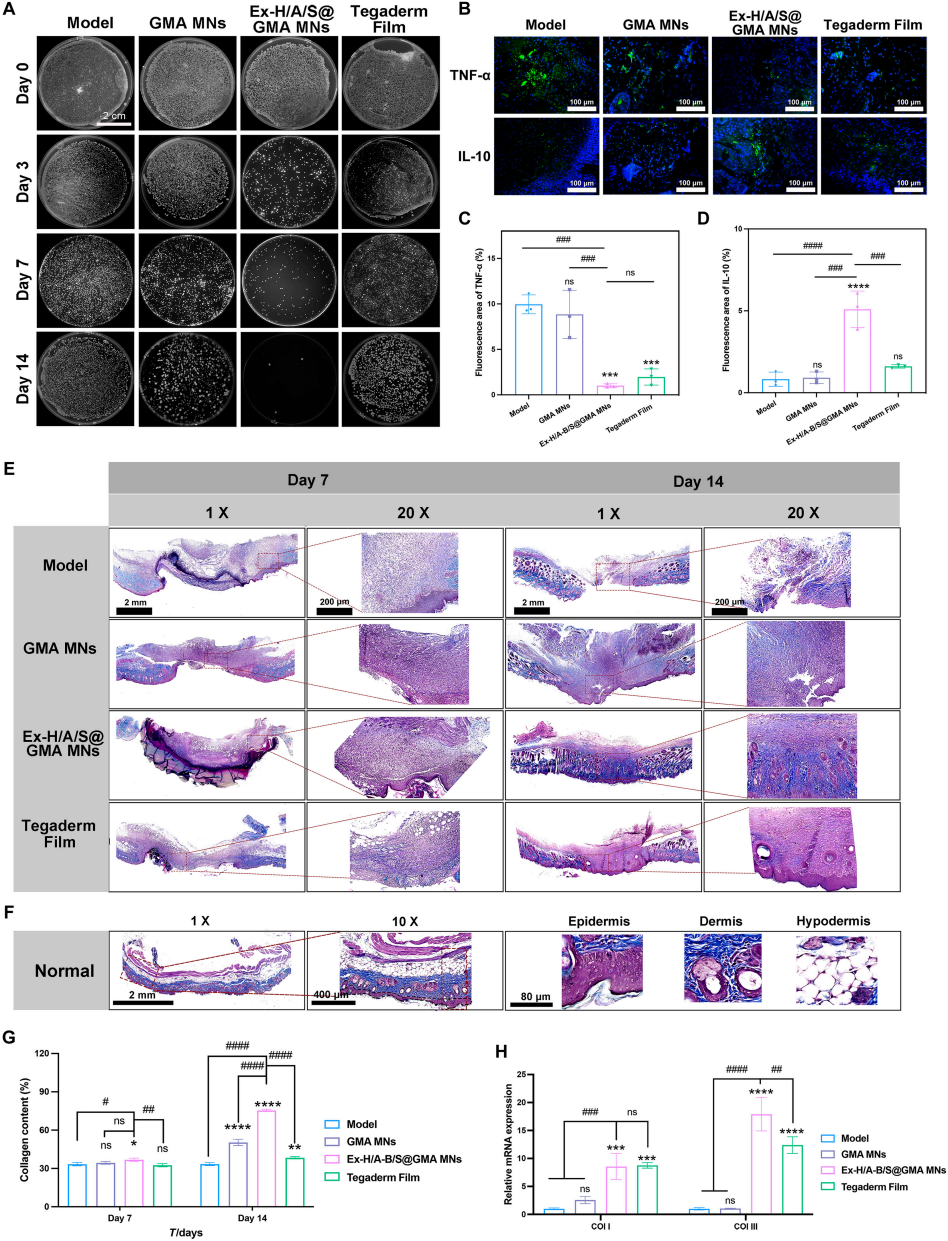
Antibacterial, anti-inflammatory, and scarless wound healing ability of Ex-H/A-B/S@GMA MNs in co-infected mice. (A) Images of survival bacteria clones. (B) TNF-α and IL-10 immunofluorescence staining (green) on day 14 and (C and D) quantitative results. (E) Masson staining. (F) Masson-stained normal skin. (G) Collagen deposition amount in regenerated skin tissues. (H) Relative mRNA expression of Col I and Col III. *n* = 3 (*P < 0.05, **P < 0.01, ***P < 0.001, ****P < 0.0001, *^#^P* < 0.05, *^##^P* < 0.01, *^###^P* < 0.001, *^####^P* < 0.0001).

The bacterial cloning experiment suggests that, by the 10th day, the antibacterial rate of Ex-H/A-B/S@GMA MN nears almost 100% (Fig. 7A and Fig. S11). The levels of TNF-α and IL-10, as characterized by fluorescence intensity, were significantly reduced by 88.6% and increased by 82.3%, respectively, following treatment with Ex-H/A-B/S@GMA MNs (Fig. 7B to D). CD206 is a marker of the surface of M2 macrophages, also known as selectively activated macrophages, and is involved in tissue repair [31]. As described in Fig. S12A and B, the CD206 positivity rate was significantly higher on day 14 in the Ex-H/A-B/S@GMA MNs treatment group, suggesting that the Ex-H/A-B/S@GMA MNs attenuated the inflammatory response. As evidenced in Fig. S12C and D, treatment with Ex-H/A-B/S@GMA MNs appears to modulate the inflammatory response, with a significant reduction in proinflammatory cytokines TNF-α, IL-1β, and IL-6, and an increase in the anti-inflammatory cytokine IL-10, suggesting a potential for promoting a balanced healing process.

The collagen fiber content of Ex-H/A-B/S@GMA MN-treated mice after 14 d, by Masson’s trichrome staining (Fig. 7E to G), was significantly elevated by 55.8%, compared to model mice, reflecting the positive role of Ex-H/A-B/S@GMA MNs in scarless wound healing in ECM deposition and collagen alignment. Figure 7H illustrates that the therapeutic treatment of Ex-H/A-B/S@GMA MNs elevated Col I and Col III expression by 88.3% and 94.4%, respectively, compared to the model group, which may be due to the release of SAB into the scarless layer. Small amounts of SAB increase cell viability, stimulate cell migration, and increase Col III expression [32,33]. Once the amount of SAB reaches a certain concentration, fibroblast migration is inhibited, which is vital in the early stages of scar formation [34,35].

### Wound healing, antibacterial, and biofilm in diabetic mice

**Fig. 8.**
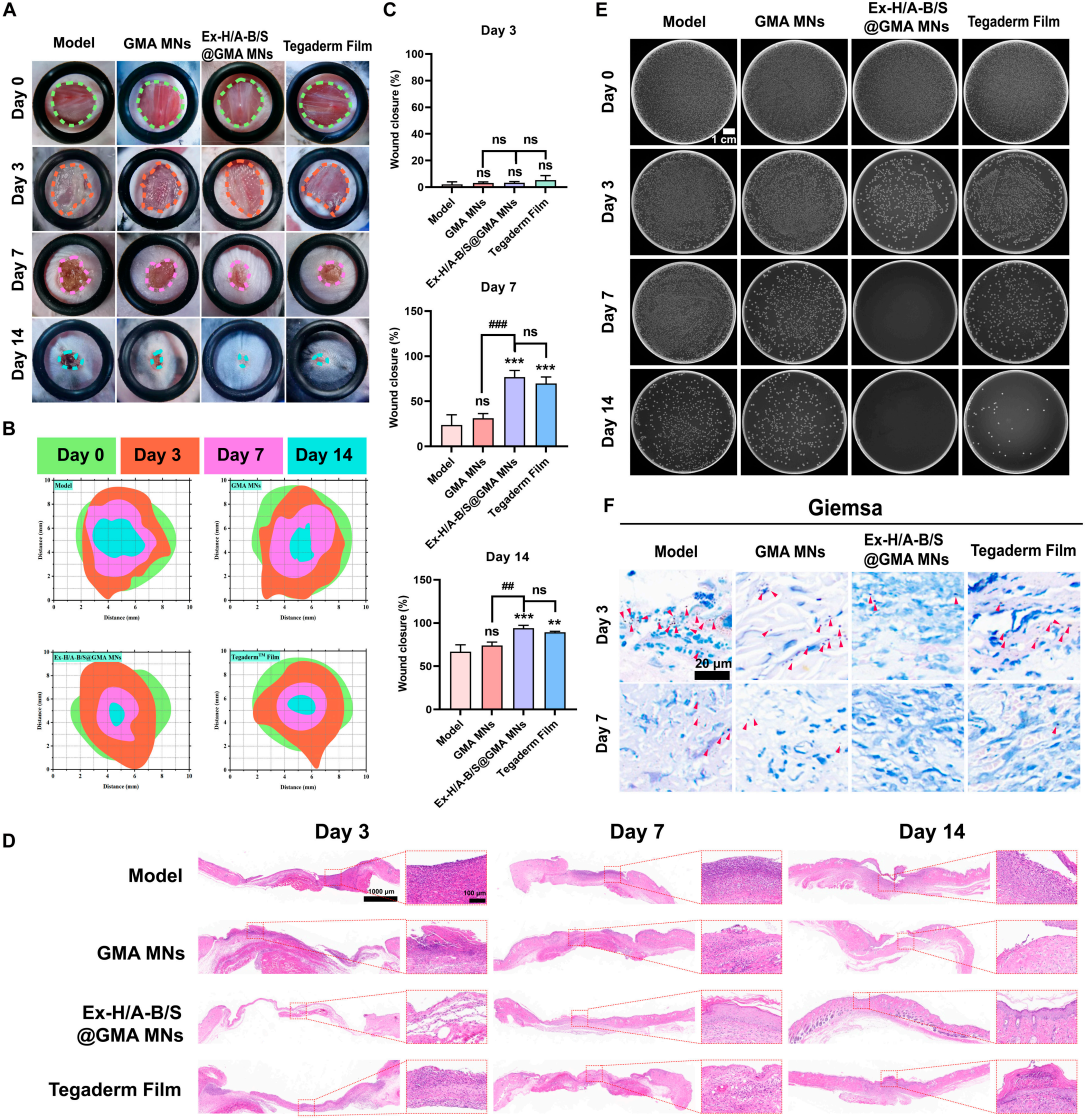
Wound healing evaluation, antibacterial, and anti-biofilm effects of Ex-H/A-B/S@GMA MNs in diabetic mice. (A) Representative photos of wounds treated with different samples. (B) Diagrammatic images of wound contraction. (C) Quantitative wound closure outcomes on days 3, 7, and 14. (D) Histological micrographs of H&E-stained sections. (E) Bacterial load on days 0, 3, 7, and 14. (F) Giemsa staining. The red triangle represents the residual bacteria. *n* = 3 (*P < 0.05, **P < 0.01, ***P < 0.001, ****P < 0.0001, *^#^P* < 0.05, *^##^P* < 0.01, *^###^P* < 0.001, *^####^P* < 0.0001).

As shown in Fig. 8A to C, on the third day of treatment, compared to the Model group and the GMA MNs group, the Ex-H/A-B/S@GMA MNs group exhibited clean wounds with no pus formation, which may be attributed to the adsorption capacity of GMA MNs and the release of AMPs with bactericidal effects. After 14 d of treatment, the Ex-H/A-B/S@GMA MNs group achieved a healing rate of nearly 100%, surpassing the other groups. The wound specimens were further stained with H&E to assess their histological status (Fig. 8D). Compared to other groups, the Ex-H/A-B/S@GMA MNs group showed less inflammatory cell infiltration after 3 d. After 7 d of treatment, a significant amount of new epidermis covered the wound, and the epidermal layer thickened. By the 14th day of treatment, the wounds in this group were nearly completely healed, with no obvious scar tissue formation. Compared to the other 3 groups, the Ex-H/A-B/S@GMA MNs group exhibited a significantly lower bacterial count, with an antibacterial effect approaching 100% (Fig. 8E and Fig. S13). This indicates that the AMPs were effectively released, demonstrating significant antimicrobial efficacy. No biofilm formation was observed on the wound surface, and the wound did not deteriorate. Further validation of biofilm formation was conducted through Giemsa staining (Fig. 8F). On day 7, almost no bacterial residue was detected in the Ex-H/A-B/S@GMA MNs group, whereas varying degrees of bacterial residue were present in the other groups, confirming the antibacterial and anti-biofilm effects of Ex-H/A-B/S@GMA MNs.

### Anti-inflammatory, anti-oxidative, angiogenesis, and scarless wound healing effects in diabetic mice

**Fig. 9.**
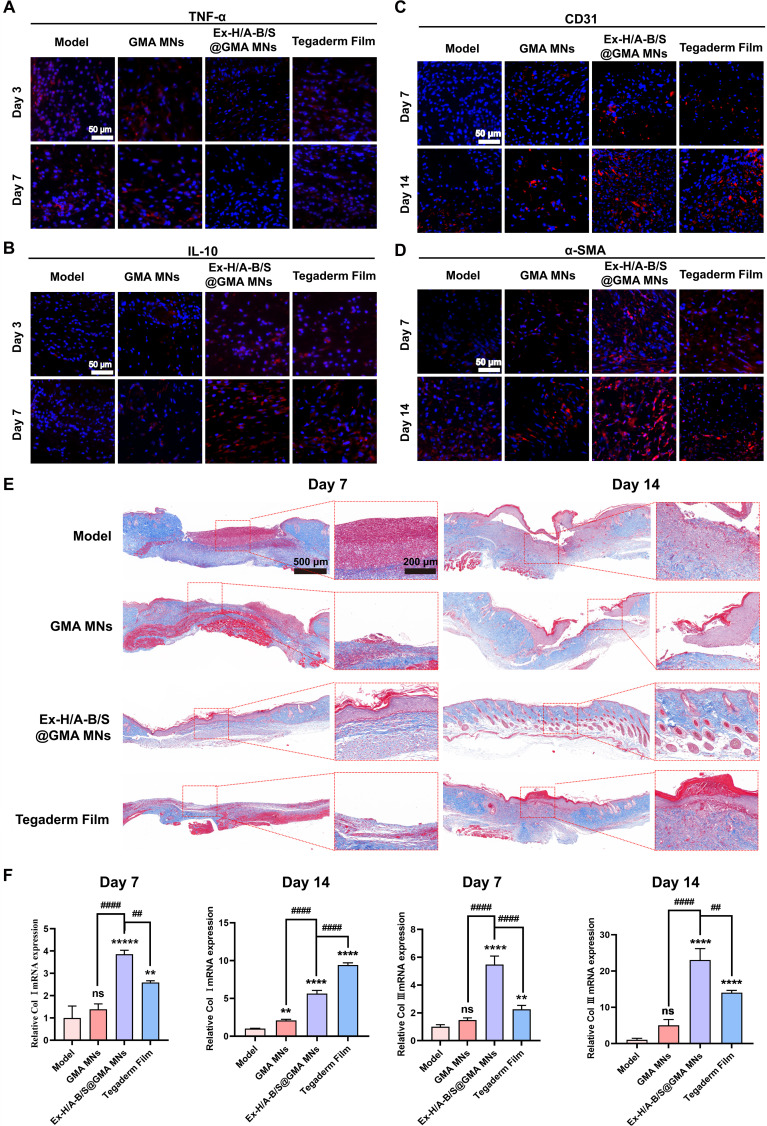
Anti-inflammatory, angiogenesis, and scarless wound healing ability of Ex-H/A-B/S@GMA MNs in diabetic mice. (A) The skin wound tissue was stained with TNF-α immunofluorescence on days 3 and 7. (B) The skin wound tissue was stained with IL-10 immunofluorescence on days 3 and 7. (C and D) Immunofluorescence staining for CD31 and α-SMA at 7 and 14 d. (E) Masson staining. (F) Relative mRNA expression of Col I and Col III at days 7 and 14. *n* = 3. An asterisk (*/^#^) indicates a statistically significant difference (**P < 0.01, ***P < 0.001, ****P < 0.0001, *^##^P* < 0.01, *^###^P* < 0.001, *^####^P* < 0.0001).

Figure S14 demonstrates that on day 3, the Ex-H/A-B/S@GMA MNs group showed reductions in pro-inflammatory cytokines (TNF-α, IL-1β, and IL-6) by 62.06%, 50.97%, and 35.42%, respectively, while the anti-inflammatory cytokine IL-10 increased by 48.92%, indicating that Ex-H/A-B/S@GMA MNs had released AMPs to exert anti-inflammatory effects. The results in Fig. 9A and B reveal that on day 3, varying degrees of expression of the inflammatory cytokine TNF-α were observed in all groups, while the expression of IL-10 was increased in the Ex-H/A-B/S@GMA MNs group. In diabetic wounds, M1 macrophages accumulate and create a damaged microenvironment characterized by oxidative stress, enhanced proteolysis, and cellular injury. Therefore, modulating macrophage polarization is a key strategy for diabetic wound healing. As shown in Fig. S15, on day 7, the Ex-H/A-B/S@GMA MNs group exhibited the highest CD206 expression. By day 14, the Ex-H/A-B/S@GMA MNs group displayed more widely distributed red fluorescent CD206-positive cells. These results indicate that Ex-H/A-B/S@GMA MNs can stimulate macrophage polarization toward the M2 phenotype, exert anti-inflammatory effects, and alter the inflammatory response.

Figure S16 shows that on days 3 and 7, compared with the model group, the MDA levels in the Ex-H/A-B/S@GMA MNs group decreased by 55.01% and 63.11%, respectively, indicating a reduction in lipid peroxidation. The CAT levels increased by 53.77% and 42.10%, respectively, suggesting an enhanced ability to scavenge hydrogen peroxide. The SOD activity increased by 52.43% and 42.41%, respectively, which helps to reduce oxidative stress by scavenging free radicals. The MPO levels decreased by 56.25% and 67.39%, respectively, indicating a reduction in neutrophils and alleviation of oxidative stress. Excess reactive oxygen species (ROS) and the corresponding oxidative microenvironment at the wound site can damage healthy epithelial cells and interfere with wound repair [36]. As shown in Fig. S17, on day 3, the Model and GMA MNs groups had severe oxidative damage, while the Ex-H/A-B/S@GMA MNs group had lower ROS levels and reduced oxidative stress. By day 7, the oxidative stress in the Model group was somewhat relieved but still significant. The GMA MNs group showed reduced inflammatory factors and ROS release due to MN adsorption. SAB’s antioxidant effects cleared free radicals, significantly reducing ROS at the wound site.

Angiogenesis is essential for wound repair, as it provides nutrients and oxygen for cell replication [37]. CD31 and α-SMA are important biomarkers of wound regeneration in endothelial cells and myofibroblasts. Figure 9C and D shows that on day 14, the expression levels of CD31 and α-SMA in the Ex-H/A-B/S@GMA MNs group were significantly increased 2.51- and 3.64-fold, respectively, compared with the model groups. This indicates that Ex-H/A-B/S@GMA MNs effectively promoted blood vessel formation and wound healing. Further verification by qPCR (Fig. S18) showed higher expression levels of CD31 and α-SMA in the Ex-H/A-B/S@GMA MNs group on days 7 and 14 compared to the Model group, confirming its significant role in promoting skin wound healing. As shown in Fig. 9E, on day 7, the Model group’s wound was not fully healed with little collagen deposition, while the Ex-H/A-B/S@GMA MNs group had thickened epidermis and more collagen deposition. On day 14, the wounds of Ex-H/A-B/S@GMA MNs were almost healed, with dense and orderly collagen and many hair follicles, whereas the film group had disordered collagen and obvious scars. Figure 9F shows that on day 7, the Ex-H/A-B/S@GMA MNs group produced more Col I and Col III, indicating better wound healing. On day 14, the film group had more scar tissue and higher Col I mRNA but lower Col III mRNA. The Ex-H/A-B/S@GMA MNs group had no evident scars and higher Col III content. Experiments showed that Ex-H/A-B/S@GMA MNs promoted collagen fibers similar to normal skin and scarless healing.

### Biosafety in vivo

Histological analysis of the major organs (heart, liver, spleen, kidney, and lungs) indicated no discernible histological changes 14 d after injection (Fig. S19A). ALT and AST concentrations were within the reference range, indicating that the Ex-H/A-B/S@GMA MNs were not hepatotoxic. CRE and BUN, which are indicators of kidney function, were also within the standard ranges, indicating no nephrotoxicity. The results show that the hydrogels have good biodegradability and biosafety (Fig. S19B).

## Conclusion

This study successfully developed a multifunctional MN patch based on the “dynamic early warning–stage intervention” strategy, aiming to achieve visual monitoring of infection and controllable drug release by responding to the wound microenvironment, providing a one-stop solution for the treatment of acute and chronic infected wounds. The patch demonstrated multiple biological activities in vitro, including hemostasis, anti-inflammatory, antibacterial, biofilm formation inhibition, and promotion of cell migration, and effectively accelerated the healing of acute wounds in co-infected mice and the repair of chronic wounds in diabetic models in animal experiments, indicating its good adaptability and therapeutic efficacy for complex wound microenvironments. The distinctive features of this study include the following: (a) Microenvironment-responsive characteristics: By sensing local physiological and biochemical changes in the wound (such as pH, temperature, or specific metabolites), dynamic regulation of drug release is achieved, avoiding dosage deviations and side effect risks associated with traditional drug delivery methods. (b) Visual monitoring capability: The patch design incorporates an early infection warning mechanism, which can indicate infection progression through intuitive methods such as color changes, providing real-time evidence for clinical intervention and contributing to the timeliness and precision of treatment. (c) Full-cycle treatment coverage: From rapid hemostasis and inflammation control for acute wounds to biofilm clearance and tissue regeneration promotion for chronic wounds, the patch can act on multiple stages of wound healing, meeting the therapeutic needs of different types of wounds. However, this study still has certain limitations: Firstly, the pathophysiological differences between animal models and human wounds may affect the clinical translation efficacy, necessitating further large animal experiments and clinical trials. Secondly, the long-term stability of the patch and the scaled production process need to be optimized. Future directions may include integrating intelligent sensors to enable remote data transmission and personalized drug delivery adjustments and developing new biodegradable substrates to reduce the risk of secondary trauma and improve patient compliance. In summary, this microenvironment-responsive MN patch provides an innovative strategy for the treatment of various types of wound infections. Its integrated “warning-intervention” design is expected to reduce the medical burden and improve patient prognosis, demonstrating significant potential for clinical translation.

## Data Availability

Data will be made available on request

## References

[1] Antimicrobial Resistance Collaborators. Global burden of bacterial antimicrobial resistance in 2019: A systematic analysis. Lancet. 2022;399(10325):629–655. 35065702 10.1016/S0140-6736(21)02724-0 PMC8841637

[2] Gouin JP, Kiecolt-Glaser JK. The impact of psychological stress on wound healing: Methods and mechanisms. Crit Care Nurs Clin North Am. 2012;24(2):201–213. 22548859 10.1016/j.ccell.2012.03.006 PMC3775570

[3] Xiong Y, Chen L, Liu P, Yu T, Lin C, Yan C, Hu Y, Zhou W, Sun Y, Panayi AC, et al. All-in-one: Multifunctional hydrogel accelerates oxidative diabetic wound healing through timed-release of exosome and fibroblast growth factor. Small. 2022;18(1): Article e2104229. 34791802 10.1002/smll.202104229

[4] Hurlow J, Bowler PG. Acute and chronic wound infections: Microbiological, immunological, clinical and therapeutic distinctions. J Wound Care. 2022;31(5):436–445. 35579319 10.12968/jowc.2022.31.5.436

[5] Jin Y, Lu Y, Jiang X, Wang M, Yuan Y, Zeng Y, Guo L, Li W. Accelerated infected wound healing by probiotic-based living microneedles with long-acting antibacterial effect. Bioact Mater. 2024;38:292–304. 38745591 10.1016/j.bioactmat.2024.05.008 PMC11091528

[6] Zhao C, Wang X, Yu L, Wu L, Hao X, Liu Q, Lin L, Huang Z, Ruan Z, Weng S, et al. Quaternized carbon quantum dots with broad-spectrum antibacterial activity for the treatment of wounds infected with mixed bacteria. Acta Biomater. 2022;138:528–544. 34775123 10.1016/j.actbio.2021.11.010

[7] Yang K, Zhou X, Li Z, Wang Z, Luo Y, Deng L, He D. Ultrastretchable, self-healable, and tissue-adhesive hydrogel dressings involving nanoscale tannic acid/ferric ion complexes for combating bacterial infection and promoting wound healing. ACS Appl Mater Interfaces. 2022;14(38):43010–43025. 36108772 10.1021/acsami.2c13283

[8] Ramalingam R, Dhand C, Mayandi V, Leung CM, Ezhilarasu H, Karuppannan SK, Prasannan P, Ong ST, Sunderasan N, Kaliappan I, et al. Core-shell structured antimicrobial nanofiber dressings containing herbal extract and antibiotics combination for the prevention of biofilms and promotion of cutaneous wound healing. ACS Appl Mater Interfaces. 2021;13(21):24356–24369. 34024104 10.1021/acsami.0c20642

[9] Kumar M, Mahmood S, Chopra S, Bhatia A. Biopolymer based nanoparticles and their therapeutic potential in wound healing—A review. Int J Biol Macromol. 2024;267(Pt 2): Article 131335. 38604431 10.1016/j.ijbiomac.2024.131335

[10] Kumar M, Keshwania P, Chopra S, Mahmood S, Bhatia A. Therapeutic potential of nanocarrier-mediated delivery of phytoconstituents for wound healing: Their current status and future perspective. AAPS PharmSciTech. 2023;24(6):155. 37468691 10.1208/s12249-023-02616-6

[11] Gupta J, Kumar D, Gupta R, Diwakar D, Shanno K, Tripathi AK, Kumar A, Kumar M. Therapeutic potential of traditional Chinese medicine loaded nanocarriers in wound management: Current status and their future perspective. Pharmacol Res Mod Chin Med. 2025;15: Article 100622.

[12] Kumar D, Pandey S, Shiekmydeen J, Kumar M, Chopra S, Bhatia A. Therapeutic potential of microneedle assisted drug delivery for wound healing: Current state of the art, challenges, and future perspective. AAPS PharmSciTech. 2025;26(1):25. 39779610 10.1208/s12249-024-03017-z

[13] Kumar M, Sethi P, Shiekmydeen J, Rastogi S, Mahmood S, Chopra S, Thomas S, Kumar D, Bhatia A. A recent review on smart sensor-integrated wound dressings: Real-time monitoring and on-demand therapeutic delivery. Int J Biol Macromol. 2025;313: Article 144251. 40381780 10.1016/j.ijbiomac.2025.144251

[14] McKenna OE, Posselt G, Briza P, Lackner P, Schmitt AO, Gadermaier G, Wessler S, Ferreira F. Multi-approach analysis for the identification of proteases within birch pollen. Int J Mol Sci. 2017;18(7): Article 1433. 28677627 10.3390/ijms18071433 PMC5535924

[15] Galli G, Saleh M. Immunometabolism of macrophages in bacterial infections. Front Cell Infect Microbiol. 2020;10: Article 607650. 33585278 10.3389/fcimb.2020.607650 PMC7879570

[16] Lin A, Liu Y, Zhu X, Chen X, Liu J, Zhou Y, Qin X, Liu J. Bacteria-responsive biomimetic selenium nanosystem for multidrug-resistant bacterial infection detection and inhibition. ACS Nano. 2019;13(12):13965–13984. 31730327 10.1021/acsnano.9b05766

[17] Efthimiou G, Tsiamis G, Typas MA, Pappas KM. Transcriptomic adjustments of Staphylococcus aureus COL (MRSA) forming biofilms under acidic and alkaline conditions. Front Microbiol. 2019;10:2393. 31681245 10.3389/fmicb.2019.02393 PMC6813237

[18] Caslin HL, Taruselli MT, Haque T, Pondicherry N, Baldwin EA, Barnstein BO, Ryan JJ. Inhibiting glycolysis and ATP production attenuates IL-33-mediated mast cell function and peritonitis. Front Immunol. 2018;9:3026. 30619366 10.3389/fimmu.2018.03026 PMC6305324

[19] Zeng ZY, Jiang GH, Liu TQ, Song G, Sun YF, Zhang XY, Jing YT, Feng MJ, Shi YF. Fabrication of gelatin methacryloyl hydrogel microneedles for transdermal delivery of metformin in diabetic rats. Bio-Des Manuf. 2021;4(4):902–911.

[20] Zandi N, Dolatyar B, Lotfi R, Shallageh Y, Shokrgozar MA, Tamjid E, Annabi N, Simchi A. Biomimetic nanoengineered scaffold for enhanced full-thickness cutaneous wound healing. Acta Biomater. 2021;124:191–204. 33508511 10.1016/j.actbio.2021.01.029

[21] Wang Y, Xiao D, Quan L, Chai H, Sui X, Wang B, Xu H, Mao Z. Mussel-inspired adhesive gelatin-polyacrylamide hydrogel wound dressing loaded with tetracycline hydrochloride to enhance complete skin regeneration. Soft Matter. 2022;18(3):662–674. 34935829 10.1039/d1sm01373d

[22] Ouyang Q, Hou T, Li C, Hu Z, Liang L, Li S, Zhong Q, Li P. Construction of a composite sponge containing tilapia peptides and chitosan with improved hemostatic performance. Int J Biol Macromol. 2019;139:719–729. 31356953 10.1016/j.ijbiomac.2019.07.163

[23] Zeng J, Wang J, Wu J, Deng R, Zhang L, Chen Q, Wang J, Jin X, Gui S, Xu Y, et al. A novel antimicrobial peptide M1-8 targets the lysosomal pathway to inhibit autolysosome formation and promote apoptosis in liver cancer cells. J Cell Mol Med. 2023;27(3):340–352. 36628597 10.1111/jcmm.17644 PMC9889723

[24] Deng R, Wu J, Zhu B, Song G, Zhou T, Yang M, Pan L, Wang J, Zou X, Lv Z, et al. Engineered exosomes loaded with M1–8 peptide for targeted therapy of hepatocellular carcinoma. Appl Mater Today. 2024;37: Article 102071.

[25] Cao L, Sun G, Zhang C, Liu W, Li J, Wang L. An intelligent film based on cassia gum containing bromothymol blue-anchored cellulose fibers for real-time detection of meat freshness. J Agric Food Chem. 2019;67(7):2066–2074. 30721049 10.1021/acs.jafc.8b06493

[26] Jablonska J, Augustyniak A, Dubrowska K, Rakoczy R. The two faces of pyocyanin—Why and how to steer its production? World J Microbiol Biotechnol. 2023;39(4):103. 36864230 10.1007/s11274-023-03548-w PMC9981528

[27] Mudaliar SB, Bharath Prasad AS. A biomedical perspective of pyocyanin from Pseudomonas aeruginosa: Its applications and challenges. World J Microbiol Biotechnol. 2024;40(3):90. 38341389 10.1007/s11274-024-03889-0 PMC10858844

[28] Teng L, Shao ZW, Bai Q, Zhang XL, He YS, Lu JY, Zou DR, Feng CL, Dong CM. Biomimetic glycopolypeptide hydrogels with tunable adhesion and microporous structure for fast hemostasis and highly efficient wound healing. Adv Funct Mater. 2021;31(43): Article 2105628.

[29] Wang Y, Wu Y, Long L, Yang L, Fu D, Hu C, Kong Q, Wang Y. Inflammation-responsive drug-loaded hydrogels with sequential hemostasis, antibacterial, and anti-inflammatory behavior for chronically infected diabetic wound treatment. ACS Appl Mater Interfaces. 2021;13(28):33584–33599. 34240605 10.1021/acsami.1c09889

[30] Wu K, Fu M, Zhao Y, Gerhard E, Li Y, Yang J, Guo J. Anti-oxidant anti-inflammatory and antibacterial tannin-crosslinked citrate-based mussel-inspired bioadhesives facilitate scarless wound healing. Bioact Mater. 2023;20:93–110. 35633874 10.1016/j.bioactmat.2022.05.017 PMC9131258

[31] Xuan X, Zhou Y, Chen A, Zheng S, An Y, He H, Huang W, Chen Y, Yang Y, Li S, et al. Silver crosslinked injectable bFGF-eluting supramolecular hydrogels speed up infected wound healing. J Mater Chem B. 2020;8(7):1359–1370. 31840731 10.1039/c9tb02331c

[32] Griffin MF, Borrelli MR, Garcia JT, Januszyk M, King M, Lerbs T, Cui L, Moore AL, Shen AH, Mascharak S, et al. JUN promotes hypertrophic skin scarring via CD36 in preclinical in vitro and in vivo models. Sci Transl Med. 2021;13(609):eabb3312. 34516825 10.1126/scitranslmed.abb3312 PMC8988368

[33] Szwedowicz U, Szewczyk A, Golab K, Choromanska A. Evaluation of wound healing activity of salvianolic acid B on in vitro experimental model. Int J Mol Sci. 2021;22(14): Article 7728. 34299351 10.3390/ijms22147728 PMC8307677

[34] Liu Q, Chu H, Ma Y, Wu T, Qian F, Ren X, Tu W, Zhou X, Jin L, Wu W, et al. Salvianolic acid B attenuates experimental pulmonary fibrosis through inhibition of the TGF-beta signaling pathway. Sci Rep. 2016;6:27610. 27278104 10.1038/srep27610 PMC4899783

[35] Chen F, Wang C, Sun J, Wang J, Wang L, Li J. Salvianolic acid B reduced the formation of epidural fibrosis in an experimental rat model. J Orthop Surg Res. 2016;11(1):141. 27852325 10.1186/s13018-016-0475-x PMC5112727

[36] Wang S, Zheng H, Zhou L, Cheng F, Liu Z, Zhang H, Wang L, Zhang Q. Nanoenzyme-reinforced injectable hydrogel for healing diabetic wounds infected with multidrug resistant bacteria. Nano Lett. 2020;20(7):5149–5158. 32574064 10.1021/acs.nanolett.0c01371

[37] Wu S, Qin B, Tang X, Cui T, Yin S, Dong H, Liu Y, Deng S, Zhang H, Feng G, et al. Enzyme-responsive microneedle patch for bacterial infection and accelerated healing of diabetic wounds. Chem Eng J. 2023;466.

