# Supplementary material for: Multifaceted Microneedle Patch: A One-Stop Solution to Combat Multitype Wound Infections
Source: Biomater Res. 2025 Dec 19;29:0290. doi: 10.34133/bmr.0290 (PMC12715103; doi:10.34133/bmr.0290)
Supplement: Supplementary 1 — Figs. S1 to S19 Table S1 [file bmr.0290.f1.docx]

**Supplementary Materials**

**Multifaceted Microneedle Patch: A One-Stop Solution to Combat Multitype Wound Infections**

**Authors**

Hui Xin^1, 2, 3,^ ^§^, Yinghua Xu^4, §^, Lingling Pan^1, 2, 3^, Shanshan Wang^4^, Bin Li^4^, Ziquan Lv^2^, Xiangjie Yao^2^, Xuan Zou^2, *^, Xiaobao Jin^3,^ ^*^, Xuemei Lu^2, 3, *^and Shuiqing Gui^1, *^

**Affiliations**

^1^ Intensive Care Unit, Shenzhen Second People's Hospital, the First Affiliated Hospital of Shenzhen University, Shenzhen 518031, People's Republic of China.

^2^ Shenzhen Center for Disease Control and Prevention, Shenzhen 518055, People's Republic of China.

^3^ Guangdong Provincial Key Laboratory of Pharmaceutical Bioactive Substances, School of Basic Medical Sciences, Guangdong Pharmaceutical University, 280 Wai Huan Dong Road, Guangzhou Higher Education Mega Center, Guangzhou 510006, People's Republic of China.

^4^ Key Laboratory of the Ministry of Health for Research on Quality and Standardization of Biotechnology Products, National Institutes for Food and Drug Control, Beijing 102629, People's Republic of China.

^§^ Contributed equally to this work.

^*^Corresponding author: Tel./Fax: +86 02039352617, E-mail address: luxuemei605@163.com.

**Supplementary Figure S1-S19**


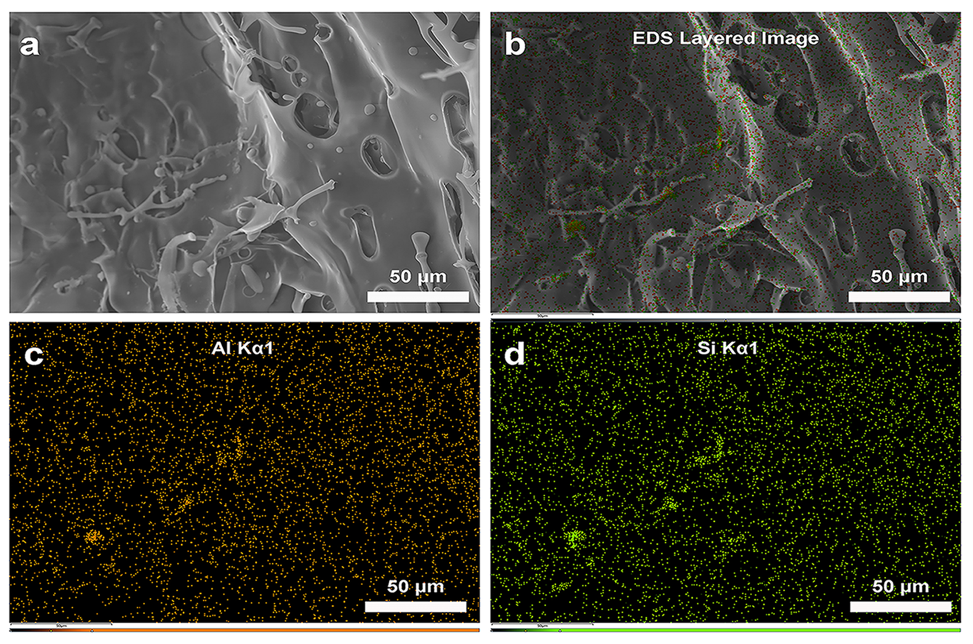


**Fig. S1.** EDS analysis of HNTs@GMA.


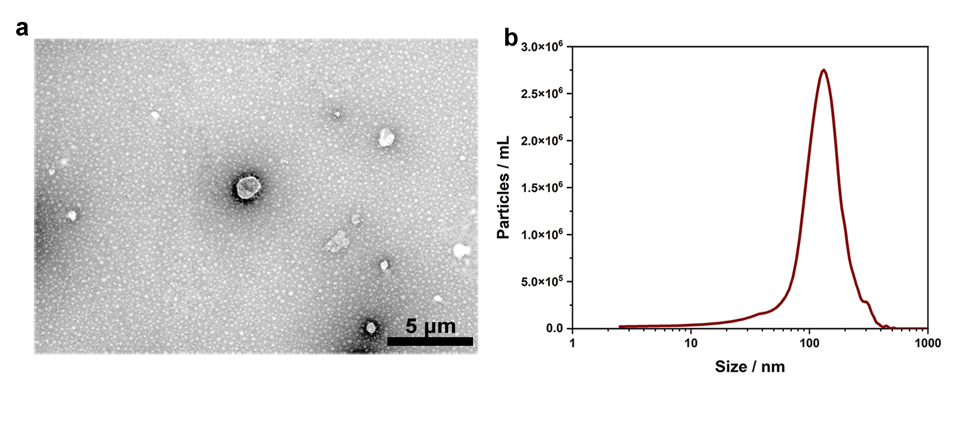


**Fig. S2.** Characterization of hBM-MSCs-Ex and the distribution of Dil-Ex in GMA.


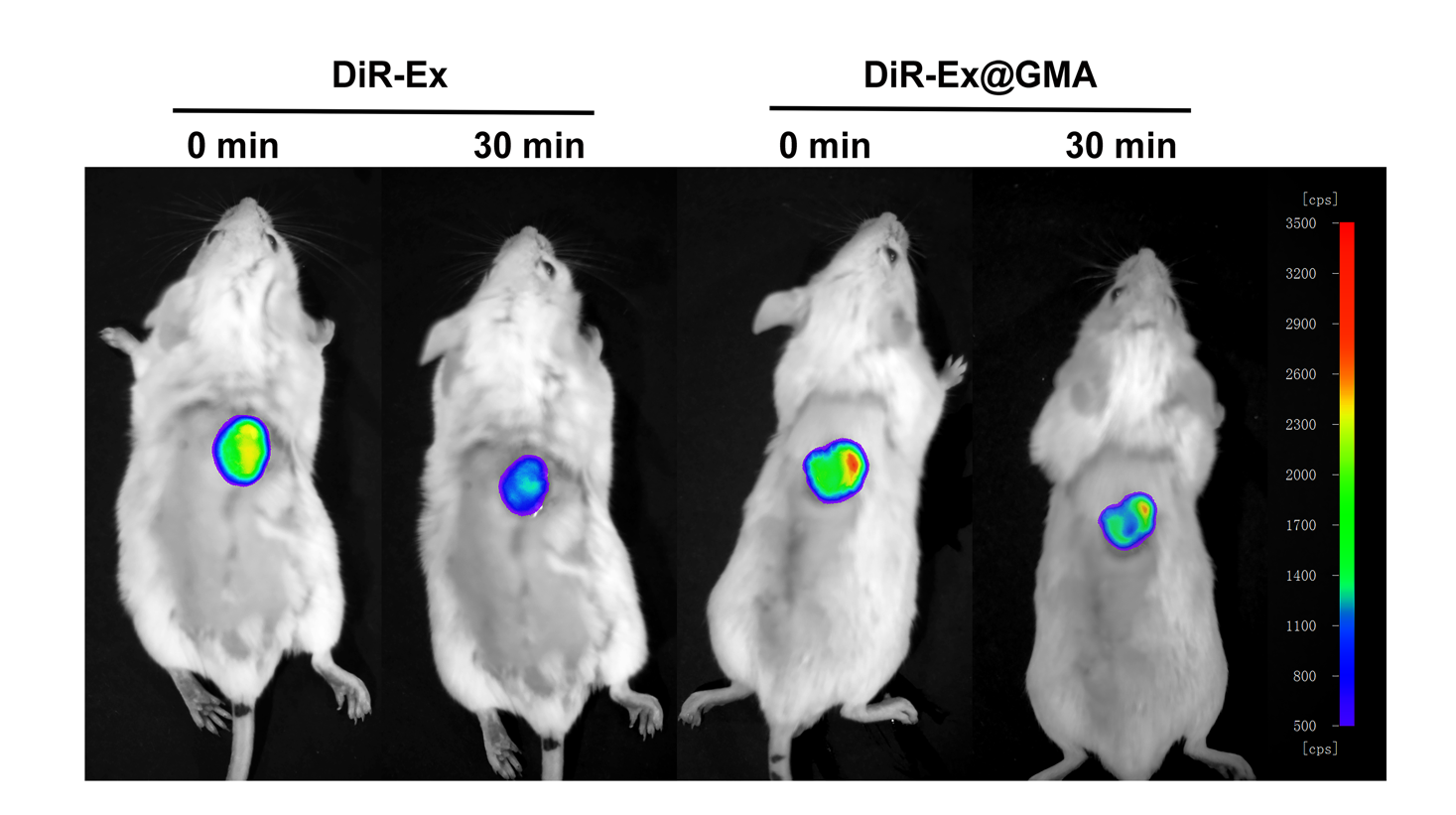


**Fig. S3.** *In vivo* imaging of Ex and Ex@GMA (0 min and 30 min).

**
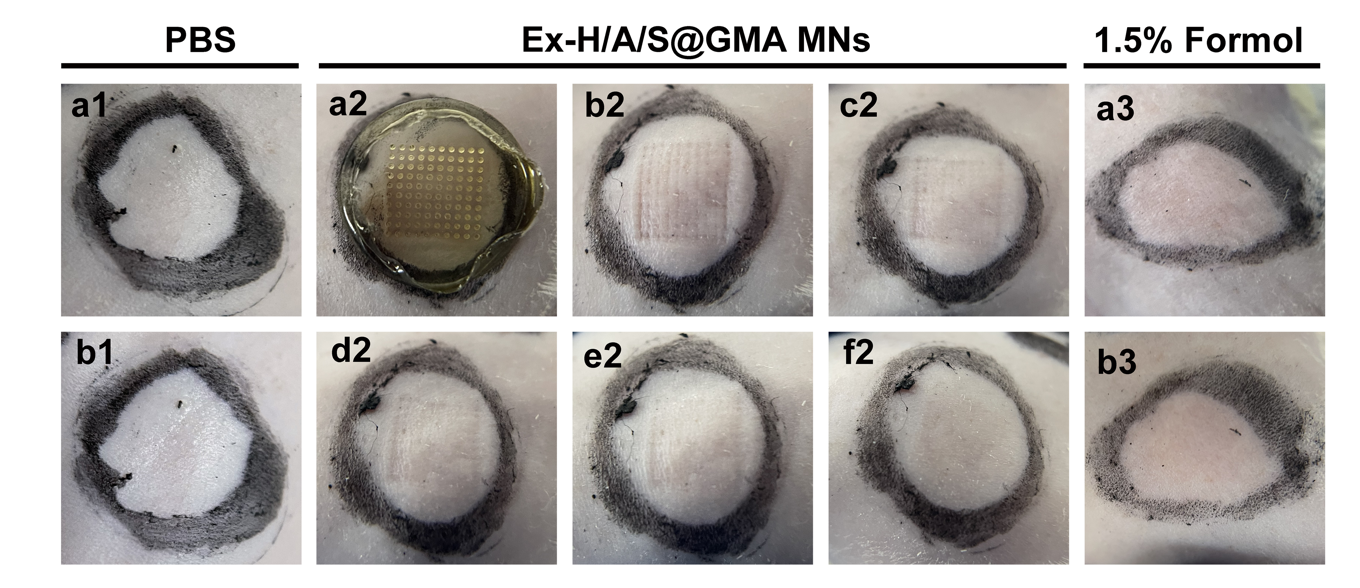
**

**Fig. S4.** **A)** Trypan blue staining penetrating the skin. **B)** Mice's skin surface changes after different treatments, PBS, Ex-H/A-B/S@GMA MNs, and 15% formol. a1: 0 min, b1: 30 min. a2-f2: 0, 5, 10, 15, 20, 30 min. a3:0 min; b3: 30 min.


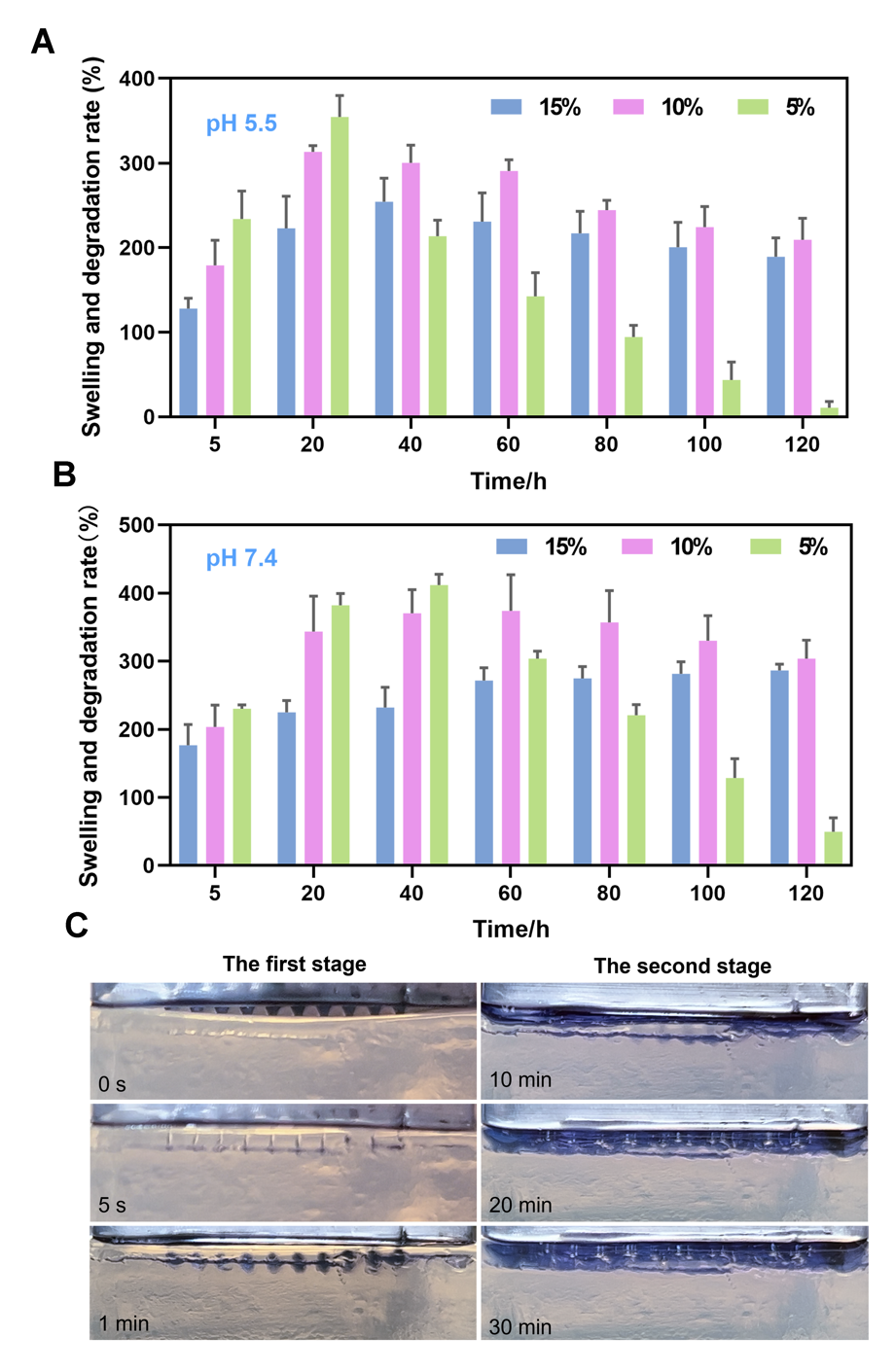


**Fig. S5. A)** GMA's swelling and degradation rate (5%, 10%, 15%) at pH 5.5. **B)** GMA's swelling and degradation rate (5%, 10%, 15%) at pH 7.4. **C)** Release process of trypan blue in artificial skin. Note: microneedles layer (10% GMA) and up layer (5% GMA).


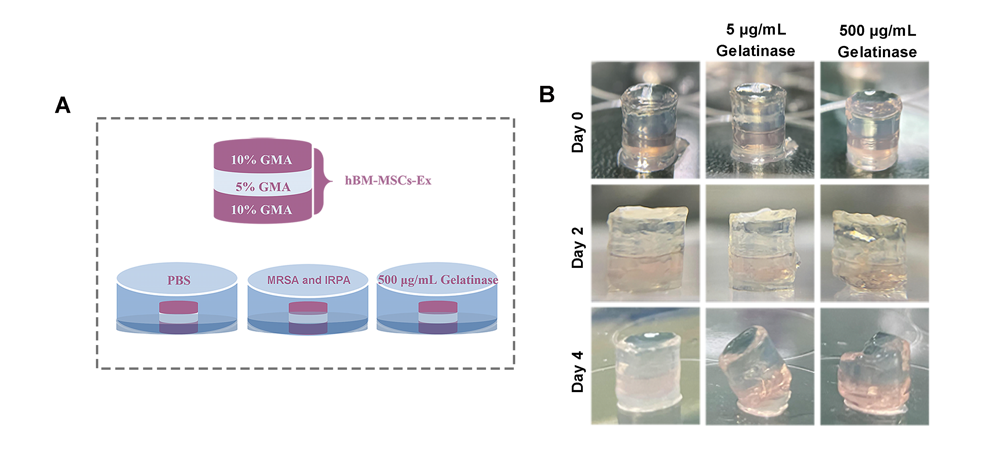


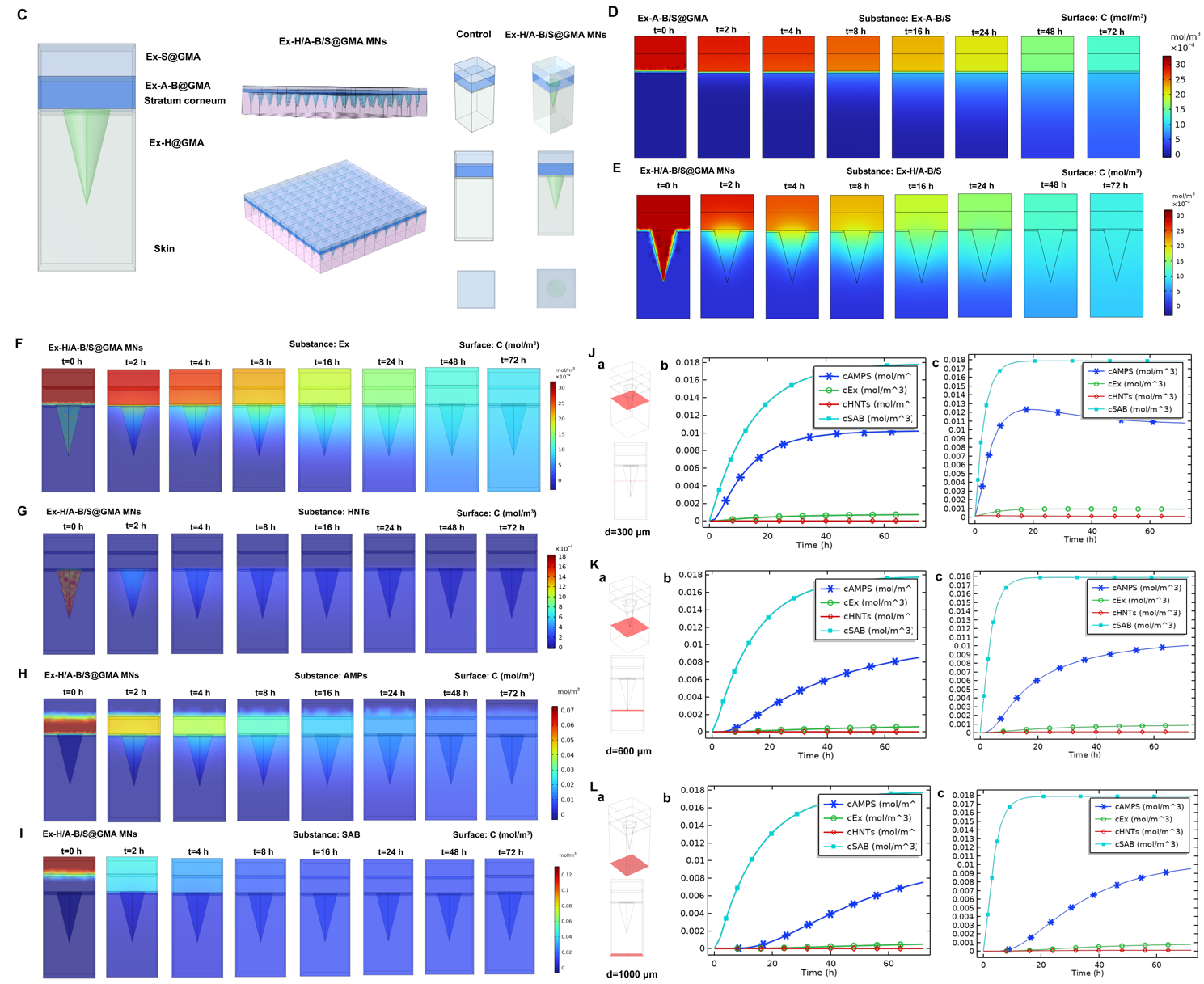


Fig. S6. Drug release studies and computational simulations. A) Three-layer structure model of swelling and degradation behavior. B) The actual swelling and degradation behavior in days 1-4. C) CAD model of the microneedle patch. D, E) Differences in drug release rates of Ex-H/A-B/S@GMA with microneedles and Ex-H/A-B/S@GMA without microneedles. F-I) Release of different substances (Ex, HNTs, AMPs, SAB) in Ex-H/A-B/S@GMA MNs. J-L) Three parallel planes (d=300, 600, 1000 μm) were used to evaluate the substance concentrations at different skin depths.


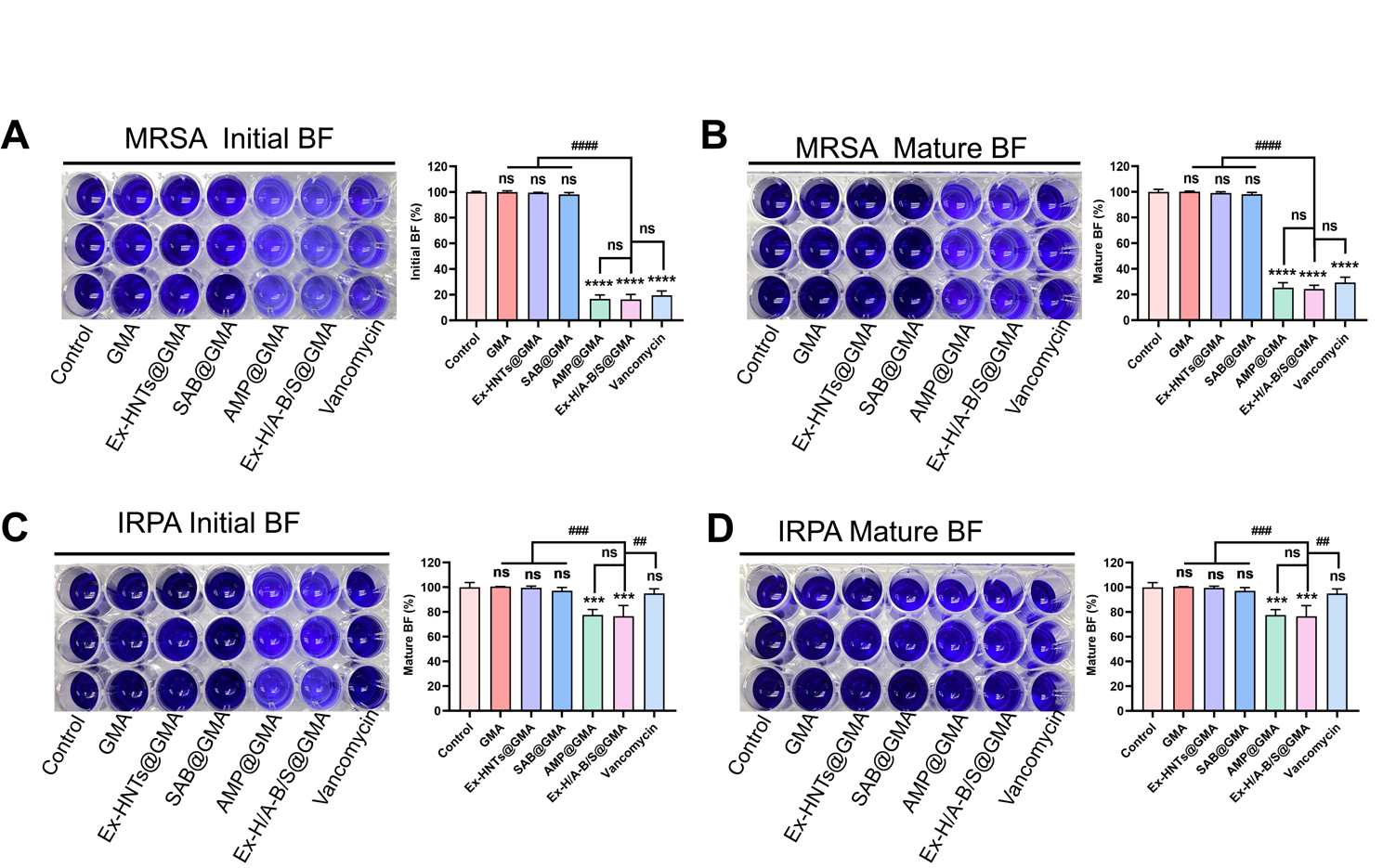


**Fig. S7.** Destruction of MRSA and IRPA by different groups on initial and mature biofilms. An asterisk (*/^#^) indicates a statistically significant difference (****p* < 0.001 and *****p* < 0.0001, *^##^p* < 0.01, *^###^p* < 0.001, *^####^p* < 0.0001).


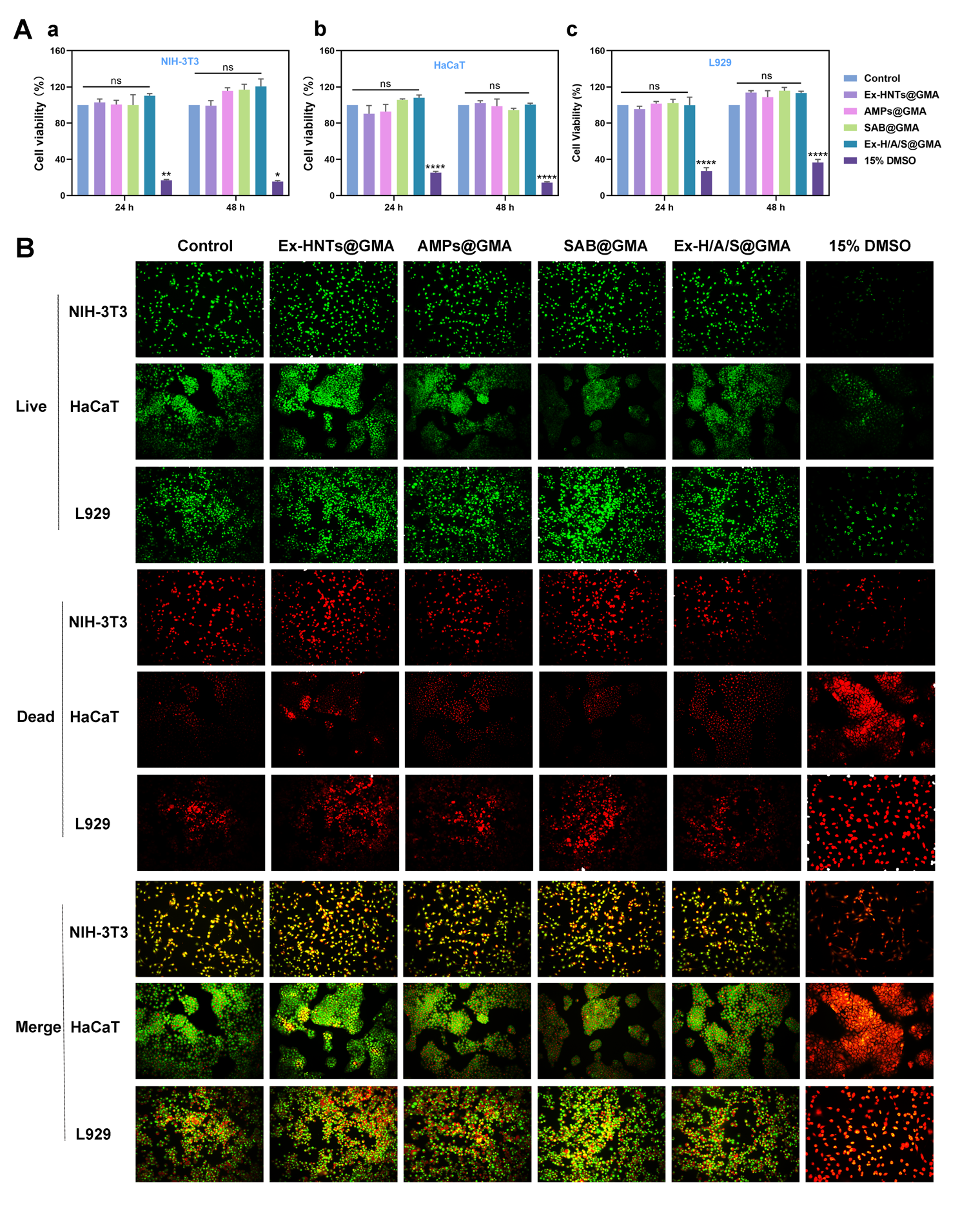


**Fig. S8.** **A)** CCK-8 results reflecting the viability of NIH-3T3, HaCaT, and L929 cells after exposure to different hydrogels of the homogenized Ex-H/A-B/S@GMA hydrogel for 24 h and 48 h. ns indicates no significance. **B)** Live/dead staining of NIH-3T3, HaCaT, and L929 cells after treating different samples for 24 h and 48 h. Calcein-AM (green/orange, for alive cells) and propidium iodide (red, for dead cells).

**Fig. S9. A)** DPPH radical scavenging efficiency of different hydrogels. **B)** Effects of different concentrations of H_2_O_2_ on NIH-3T3 cell viability. **C)** Protective effects of different hydrogels on NIH-3T3 cells under H_2_O_2_. **D)** SOD activity of cells protected by different hydrogels was measured. Note: **p* < 0.05, ***p* < 0.01, ****p* < 0.001, *****p* < 0.0001; ^#^*p* < 0.05, ^##^*p* < 0.01, ^###^*p* < 0.001, ^####^*p* < 0.0001, ns indicates no significant difference.


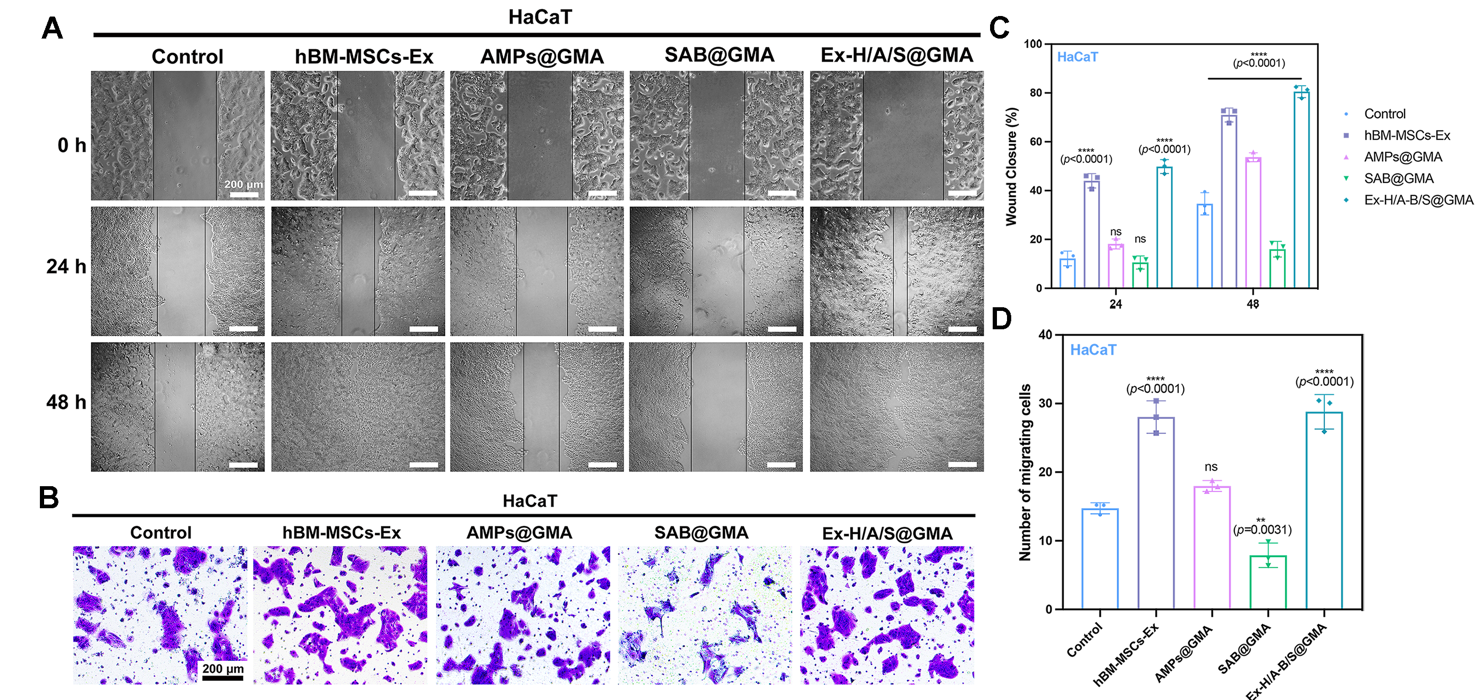


**Fig. S10.** **A)** Photographs and **B)** quantitative results of HaCaT cell migration over time. **C)** Transwell migration assays and **D)** quantitative analysis of the number of migratory cells were performed on HaCaT cells with different treatments.


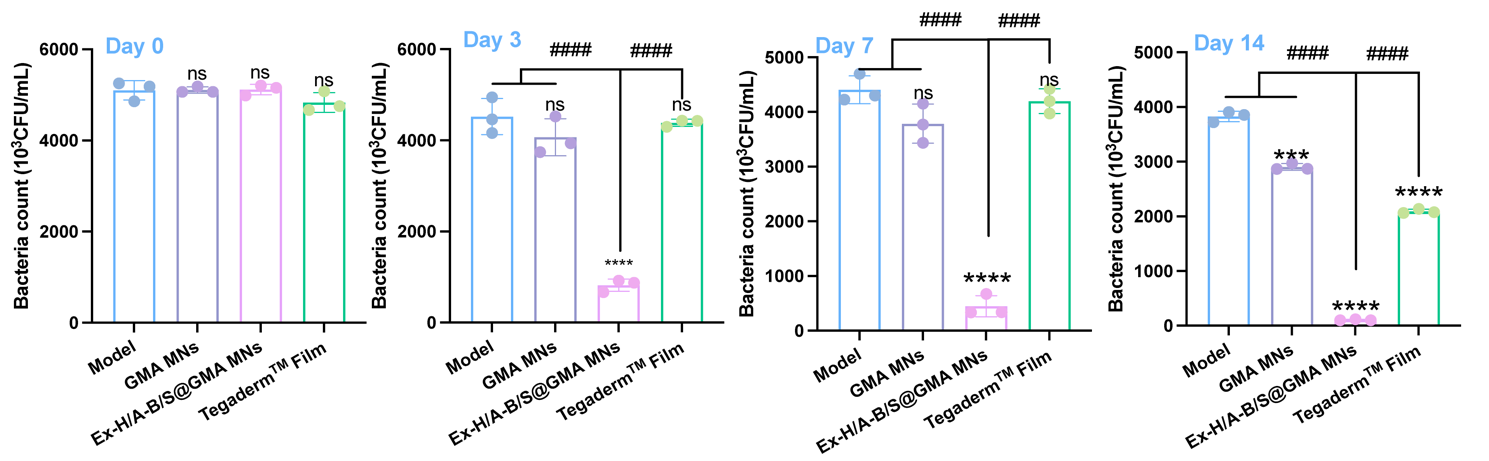


**Fig. S11.** Bacterial numbers in different groups on days 0, 3, 7, and 14 *in vivo*.

*
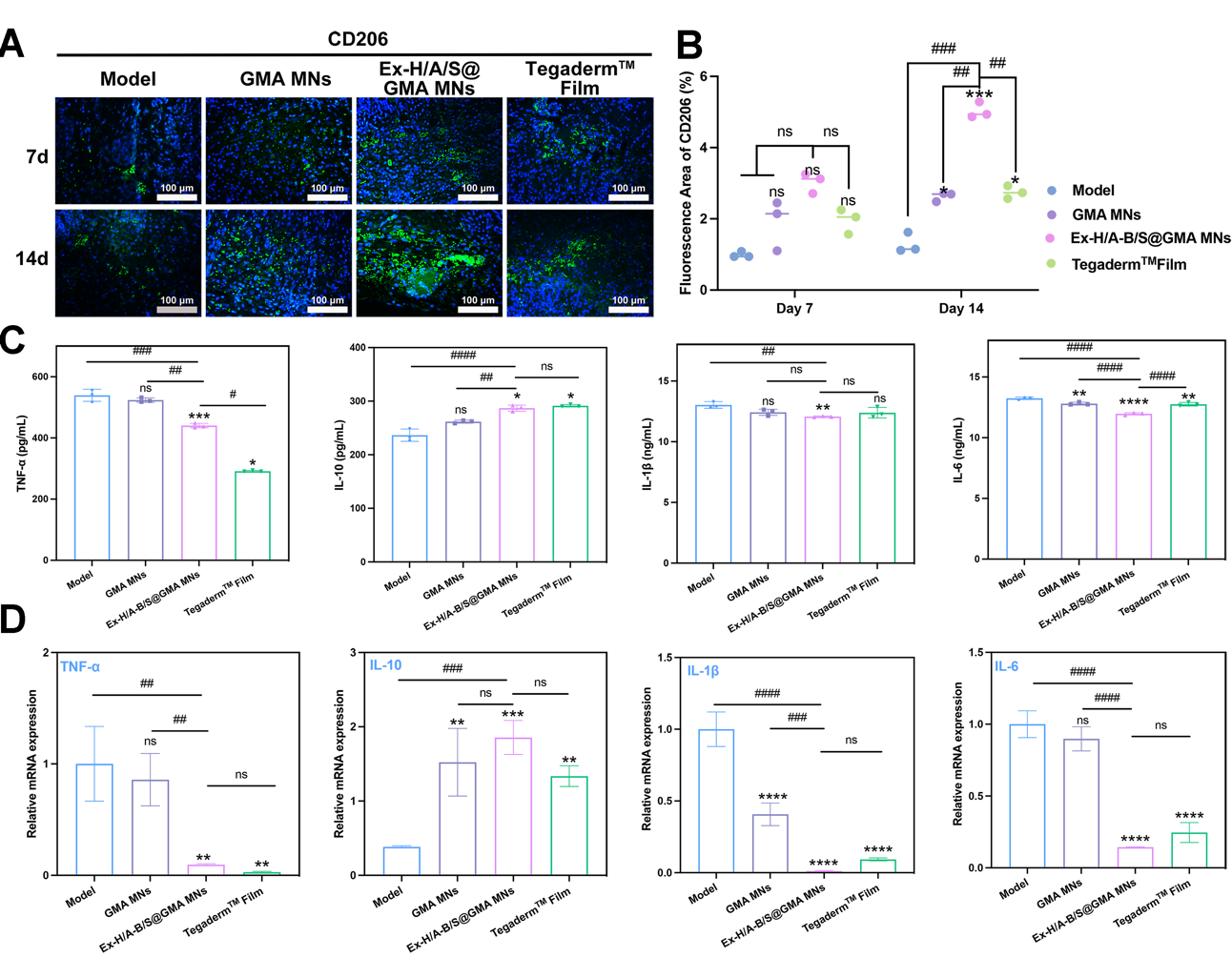
*

**Fig. S12. A)** CD206 immunofluorescence staining (green) and **B)** quantitative results. **C)** TNF-α, IL-10, IL-1β, and IL-6 ELISA results on day 3. **D)** TNF-α, IL-10, IL-1β, and IL-6 qPCR results on day 3. An asterisk (*/^#^) indicates a statistically significant difference (**p* < 0.05, ***p* < 0.01, ****p* < 0.001 and *****p* < 0.0001, *^#^p* < 0.05, *^##^p* < 0.01, *^###^p* < 0.001, *^####^p* < 0.0001).


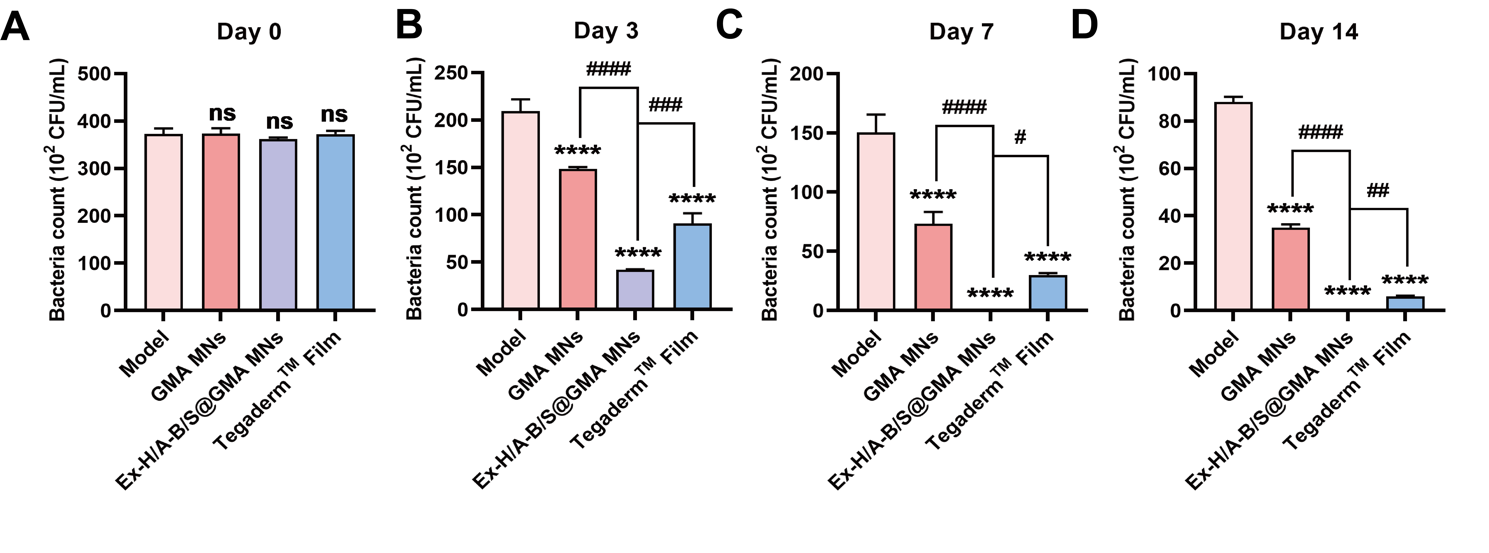


**Fig. S13.** Colonies were counted on different days in diabetic mice.


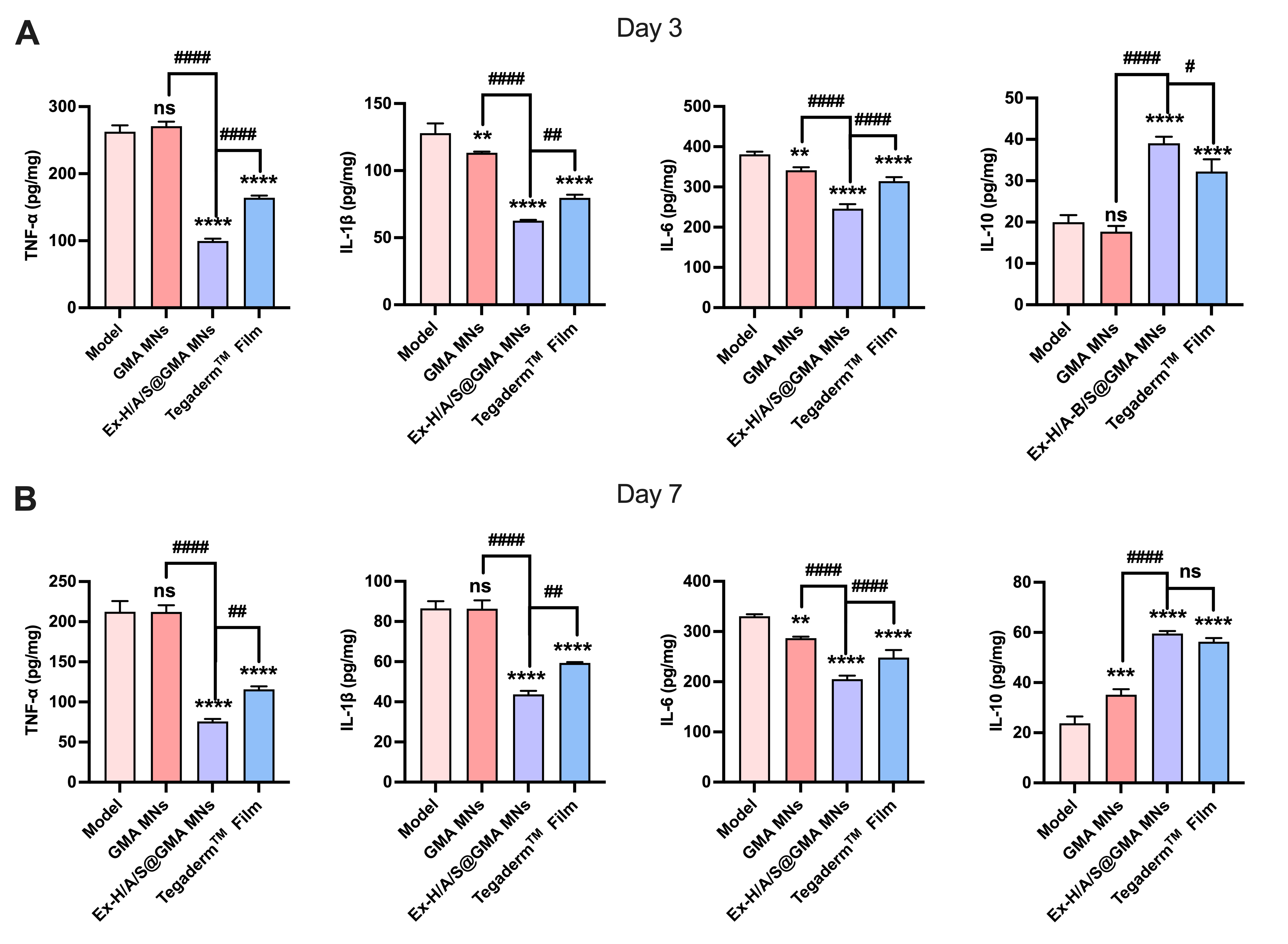


**Fig. S14. A, B)** Skin inflammatory factors on day 3 and 7 were determined by Elisa. Note: **p* < 0.05, ***p* < 0.01, ****p* < 0.001, *****p* < 0.0001; ^#^*p* < 0.05, ^##^*p* < 0.01, ^###^*p* < 0.001, ^####^*p* < 0.0001, ns indicates no significant difference.

**Fig. S15.** Skin wound tissue was stained with CD206 immunofluorescence at 7 and 14 days.


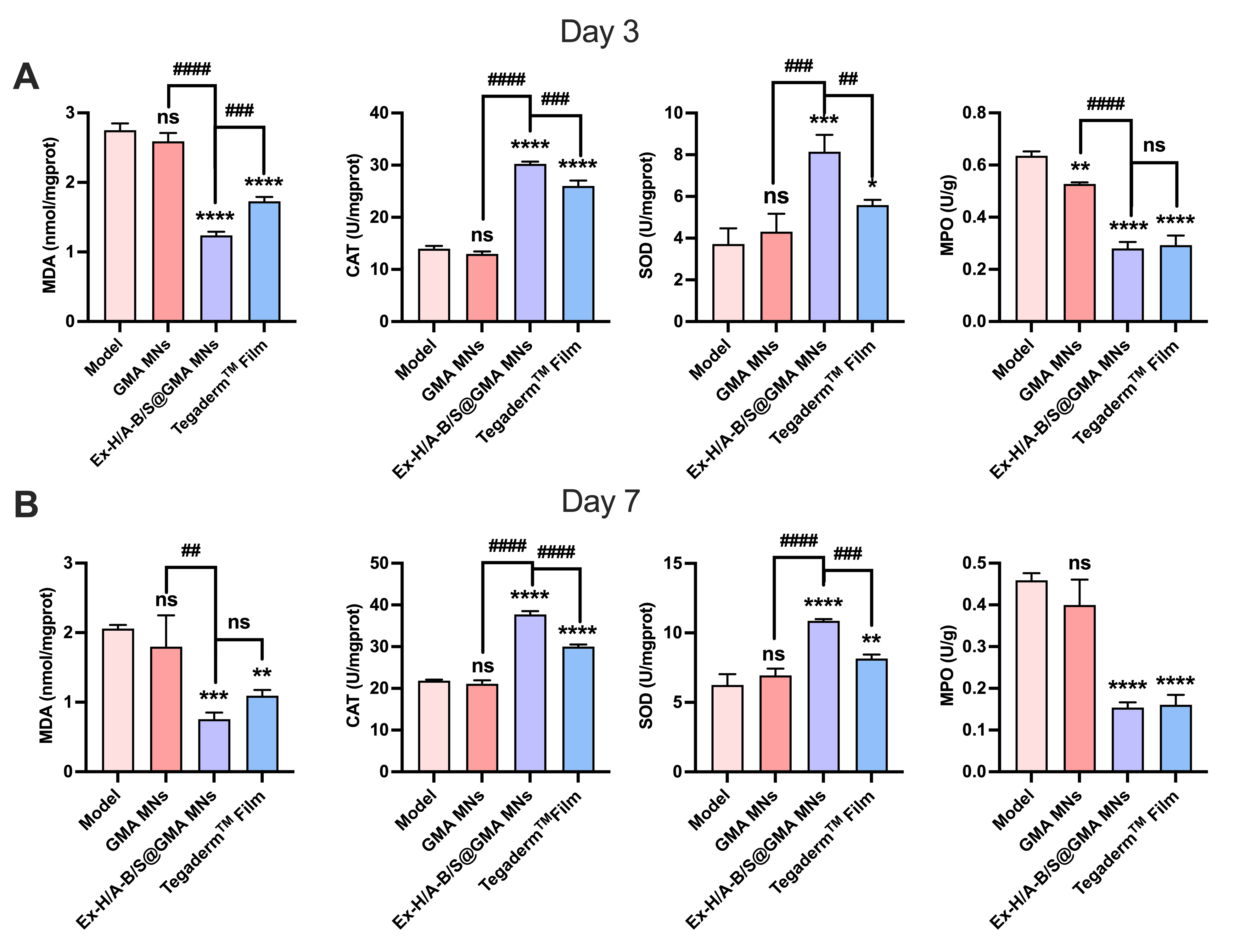


**Fig. S16. A-H)** Assessment of oxidative stress markers at 3 and 7 days.

**Fig. S17.** **A)** DHE staining analysis of skin tissue at 3 and 7 days. Note: **p* < 0.05, ***p* < 0.01, ****p* < 0.001, *****p* < 0.0001; ^#^*p* < 0.05, ^##^*p* < 0.01, ^###^*p* < 0.001, ^####^*p* < 0.0001, ns indicates no significant difference.


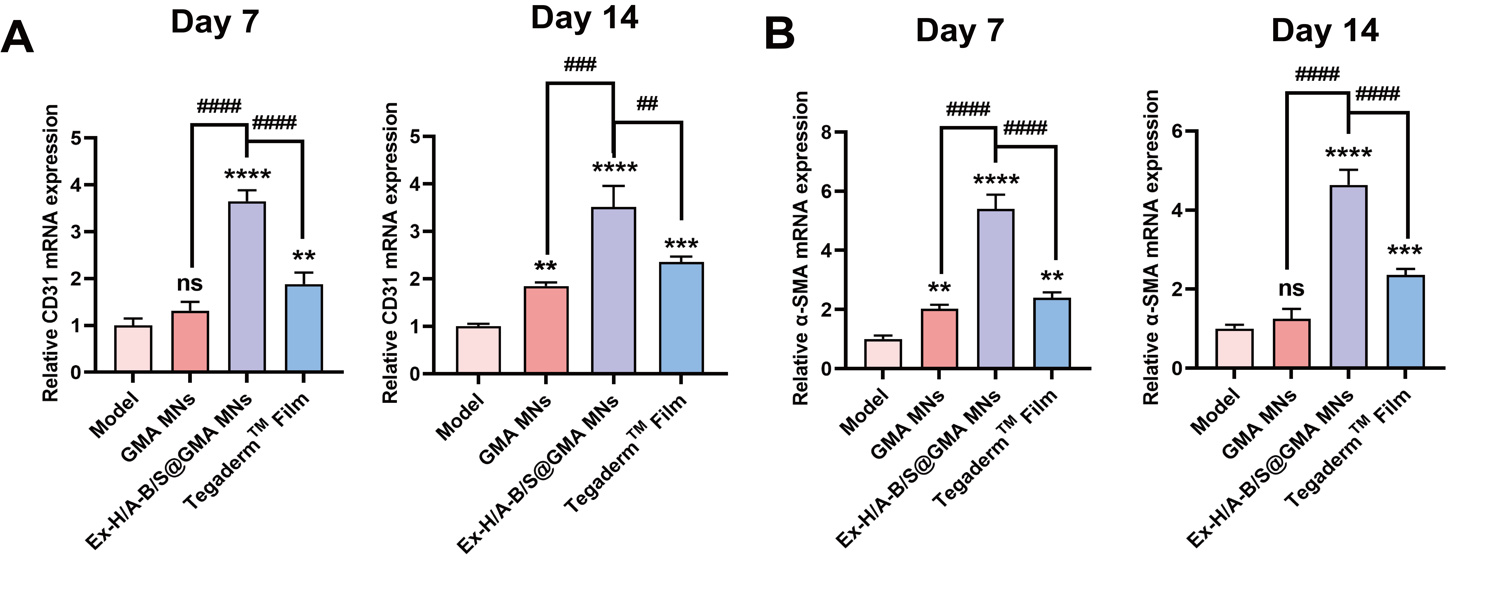


**Fig. S18.** **A**) Relative mRNA expression of CD31 at days 7 and 14. **B**) Expression levels of α-SMA mRNA after 7 and 14 days. An asterisk (*/^#^) indicates a statistically significant difference (***p* < 0.01, ****p* < 0.001 and *****p* < 0.0001, *^##^p* < 0.01, *^###^p* < 0.001, *^####^p* < 0.0001).


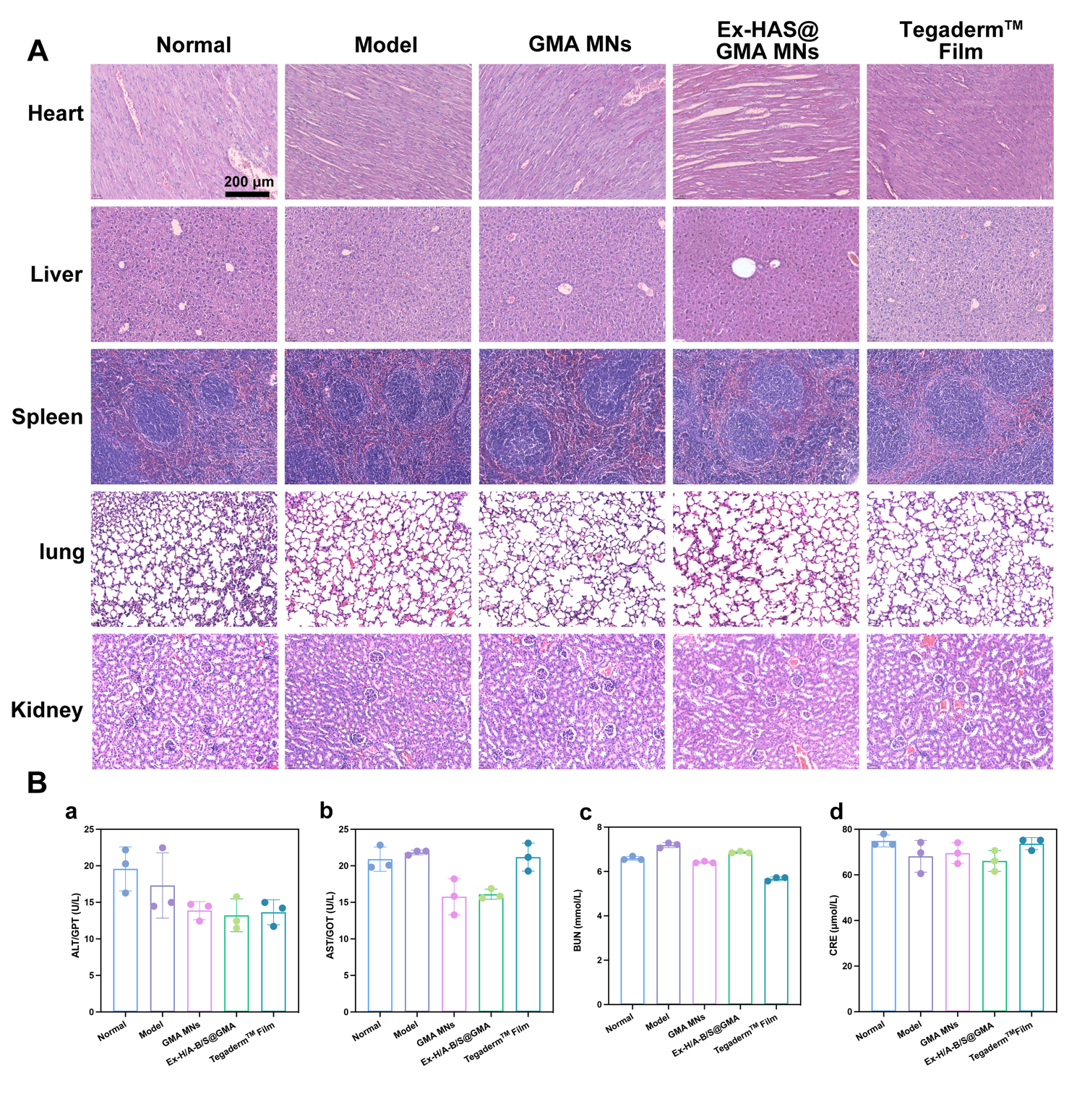


**Fig. S19. A)** Toxicological histological observation of H&E staining in the heart, liver, spleen, lungs, and kidneys with different treatment regimens. **B)** ALT, AST, CREA, and BUN liver function assays.

Table S1. Model parameters and initial drug concentrations

| **Name** | **Expression** | **Value** | **Note** |
| --- | --- | --- | --- |
| a | 1000[μm] | 0.001 m | Skin length |
| b | 600[μm] | 6E-4 m | Skin width |
| r | 150[μm] | 1.5E-4 m | Microneedle radius |
| c | 600[μm] | 6E-4 m | Microneedle height |
| d | 600[μm] | 6E-4 m | Microneedle patch width |
| e | 150[μm] | 1.5E-4 m | Microneedle patch length |
| D1 | 1.58e^-11^[m^2^/s] | 1.58E-11 m²/s | Macromolecules in microneedle patch |
| D2 | 8.3e^-12^[m^2^/s] | 8.3E-12 m²/s | Macromolecules in microneedle patch |
| D3 | 1.2e^-13^[m^2^/s] | 1.2E-13 m²/s | Macromolecules in the cuticle |
| D4 | 5e-14[m^2^/s] | 5E-14 m²/s | Macromolecules in the cuticle |
| D5 | 8.93e^-11^[m^2^/s] | 8.93E-11 m²/s | Macromolecules in the epidermis/dermis |
| D6 | 1.58e^-12^[m^2^/s] | 1.58E-12 m²/s | Macromolecules in the epidermis/dermis |
| c1 | 6.86e^-2^[mol/m^3^] | 0.0686 mol/m³ | AMPs |
| c2 | 2.96e^-3^[mol/m^3^] | 0.00296 mol/m³ | Ex |
| c3 | 2.96e^-3^[mol/m^3^] | 0.00296 mol/m³ | HNTs |
| c4 | 1.25e^-1^[mol/m^3^] | 0.125 mol/m³ | SAB |
